# Unravelling the genetic diversity and population structure of common walnut in the Iranian Plateau

**DOI:** 10.1186/s12870-023-04190-2

**Published:** 2023-04-18

**Authors:** Robabeh Shahi Shavvon, Hai-Ling Qi, Mohammad Mafakheri, Pen-Zheng Fan, Hong-Yu Wu, Fatemeh Bazdid Vahdati, Hanady S. Al-Shmgani, Yue-Hua Wang, Jie Liu

**Affiliations:** 1 Department of Biology, Faculty of Science, Yasouj University, Yasouj, Iran; 2 CAS Key Laboratory for Plant Diversity and Biogeography of East Asia, Kunming Institute of Botany, Chinese Academy of Sciences, Kunming, 650201 Yunnan China; 3 Germplasm of Bank of Wild Species, Kunming Institute of Botany, Chinese Academy of Sciences, Kunming, 650201 Yunnan China; 4 School of Ecology and Environmental Science, Yunnan University, Kunming, 650091 China; 5 Department of Plant Sciences, University of California - Davis, Davis, CA 95616 USA; 6 University of Chinese Academy of Sciences, Beijing, 100049 China; 7 Department of Biology, Faculty of Science, University of Guilan, Rasht, Iran; 8 Department of Biology, College of Education for Pure Sciences (Ibn Al-Haitham), University of Baghdad, Baghdad, Iraq

**Keywords:** Common walnut, Germplasm conservation, Genetic variation, Iranian Plateau, Persian walnut, Population structure, Refugia

## Abstract

**Background:**

Common walnut (*Juglans regia* L.) has a long cultivation history, given its highly valuable wood and rich nutritious nuts. The Iranian Plateau has been considered as one of the last glaciation refugia and a centre of origin and domestication for the common walnut. However, a prerequisite to conserve or utilize the genetic resources of *J. regia* in the plateau is a comprehensive evaluation of the genetic diversity that is conspicuously lacking. In this regard, we used 31 polymorphic simple sequence repeat (SSR) markers to delineate the genetic variation and population structure of 508 *J. regia* individuals among 27 populations from the Iranian Plateau.

**Results:**

The SSR markers expressed a high level of genetic diversity (*H*_O_ = 0.438, and *H*_E_ = 0.437). Genetic differentiation among the populations was moderate (*F*_ST_ = 0.124), and genetic variation within the populations (79%) significantly surpassed among populations (21%). The gene flow (*N*_m_ = 1.840) may have remarkably influenced the population genetic structure of *J. regia*, which can be attributed to anthropological activities and wind dispersal of pollen. The STRUCTURE analysis divided the 27 populations into two main clusters. Comparing the neighbor-joining and principal coordinate analysis dendrograms and the Bayesian STRUCTURE analysis revealed the general agreement between the population subdivisions and the genetic relationships among the populations. However, a few geographically close populations dispersed into different clusters. Further, the low genetic diversity of the Sulaymaniyah (SMR) population of Iraq necessitates urgent conservation by propagation and seedling management or tissue culture methods; additionally, we recommend the indispensable preservation of the Gonabad (RGR) and Arak (AKR) populations in Iran.

**Conclusions:**

These results reflected consistent high geographical affinity of the accession across the plateau. Our findings suggest that gene flow is a driving factor influencing the genetic structure of *J. regia* populations, whereas ecological and geological variables did not act as strong barriers. Moreover, the data reported herein provide new insights into the population structure of *J. regia* germplasm, which will help conserve genetic resources for the future, hence improving walnut breeding programs’ efficiency.

**Supplementary Information:**

The online version contains supplementary material available at 10.1186/s12870-023-04190-2.

## Background

Genetic diversity has been constantly found to be essential for adapting the population to environmental changes [1]. Genetic diversity analysis of plants' germplasm can expand our knowledge of evolution and genetic variability determinants giving profound insights into plant conservation [1-3].

*Juglans* L. belongs to the Juglandaceae family, which includes ca. 21 deciduous tree species [4]; It is a widespread genus ranging from North and South America, the West Indies, and Southeast Europe to East Asia [4-6]. *Juglans regia* L., also called common walnut, English walnut, or Persian walnut (hereafter refer as common walnut), is monoecious and heterodichogamous with 2n = 2x = 32 [7]. The species is cultivated across the temperate and tropical regions of the world for its high-quality timber and for its excellent, and edible nuts [8, 9]. High genetic variation has been reported among walnut populations worldwide, and seed reproduction, high heterozygosity, and allogamy have been suggested to be responsible for the shaping of high genetic differentiation in this species [10-12].

Common walnut is one of the most essential fruit trees cultivated primarily for edible nuts since ancient times. It is highly supported that walnut originated from the Iranian Plateau [13-15]; previous findings proposed that the walnut’s domestication first occurred in the plateau, including parts of Iran, the southern Caucasus, Turkmenistan, and afterward expanded east to China and west to Europe by human movement via the Persian Royal Road and Silk Road [14, 16-18].

The Iranian Plateau is situated at the upper plate of the Arabia-Eurasia collision zone [19]. The plateau is located between East Azerbaijan province to the northwest of Iran (East Azerbaijan province), and Afghanistan and Pakistan to the east, and Zagros Mountains to the west. It also includes the Kurdistan region of Iraq, Turkmenistan, and smaller parts of the Republic of Azerbaijan. The plateau hosts an extraordinarily diverse flora given the specific topographic conditions, such as heterogeneous landscapes [20]. The largest part of the plateau is in Iran (almost 64%), where *J. regia* cultivation is 4.48 × 10^4^ ha, producing 3.57 × 10^5^ tons of nuts in shell, and is ranked the third globally [21].

*Juglans regia* formed fragmented forests in the west of Asia during the Pleistocene [22]. It has been revealed [23, 24] that *Juglans* pollen was present throughout the Upper Paleolithic in Georgia and northwestern Iraq. Glacial refugia often show more values of genetic diversity conservation than postglacially colonized regions [25]. The Iranian Plateau has been considered as one of the ancient centres of diversity and an important glacial refugium of the common walnut after Pleistocene glaciations in Asia [22].

The Iranian Plateau contains several mountain ranges, such as the Alborz, Zagros, Kopet Dagh, Baluchistan, and Hindu Kush, etc.; hence, they might have facilitated the genetic divergence of *J. regia* populations. Nowadays, the walnut in the plateau is distributed in different ecological environments and geographic conditions. As elevation increases, climate, and physicochemical properties are also affected, which may increase population variation. These conditions might support the potential of the Iranian Plateau walnut as a rich gene resource.

Simple sequence repeats (SSRs) or microsatellites, due to their codominant inheritance, being genome- and locus-specific, high polymorphism [26, 27], have been widely utilized in studying of genetic variation of common walnut [21, 28-41]. However, they all focused on evaluating the genetic variability of regional genotypes with a few markers and populations. Additionally, political instability in some countries, such as Iraq and Afghanistan, and formidable geographic barriers to sampling have made little information available for the whole plateau. Due to the widespread distribution of *J. regia* and the lack of an accurate, comparative, and large-scale molecular study, the extent and structure of the *J. regia* genetic diversity across the Iranian Plateau have never been undertaken. To fill the gap, based on a comprehensive population-level field sampling of common walnut from four countries across the Iranian Plateau, we aim to 1) evaluate the genetic diversity of walnut populations using microsatellites, 2) assess the genetic differentiation and structure of walnut populations, and 3) propose the recommendations for germplasm conservation and resource development and utilization.

## Results

### Genetic diversity of SSR markers

All the primers produced polymorphic fragments where the average number of alleles across marker loci was 6.129 and ranged from 3 (in JM5446) to 11 (in JS12) (Table 1). The number of effective alleles (*N*_E_) varied from 1.024 (JM5446) to 3.964 (BFU-Jr38), with an average of 2.379. Observed heterozygosity (*H*_O_) and expected heterozygosity (*H*_E_) varied from 0.012 (JM5446) and 0.023 (JM5446) to 0.697 (JS09) and 0.744 (JS12), with a mean of 0.437 and 0.511, respectively.

**Table 1:** Genetic diversity of the 31 microsatellite loci used in this study

Locus	*N*_A_	*N*_E_	*I*	*H*_O_	*H*_E_	*uH*_E_	*F*	*N*_m_	*PIC*
JR02	7	2.028	1.020	0.490	0.507	0.507	0.033	2.034	0.498
JR03	6	1.519	0.605	0.280	0.342	0.342	0.181	1.044	0.346
JR04	8	3.014	1.292	0.610	0.668	0.669	0.087	2.448	0.670
JR05	7	1.517	0.661	0.334	0.341	0.341	0.020	1.834	0.346
JR06	4	2.036	0.938	0.442	0.509	0.509	0.131	1.515	0.518
JR07	4	2.518	1.017	0.565	0.603	0.603	0.063	2.248	0.606
JR08	5	1.073	0.176	0.057	0.068	0.068	0.170	1.155	0.065
JR09	4	1.896	0.795	0.362	0.473	0.473	0.234	1.253	0.477
JR10	4	1.919	0.913	0.406	0.479	0.479	0.152	1.354	0.479
JR11	8	1.721	0.850	0.369	0.419	0.419	0.118	1.646	0.420
JR12	7	3.511	1.409	0.610	0.715	0.716	0.147	1.499	0.713
JS02	6	1.494	0.629	0.310	0.331	0.331	0.062	1.508	0.335
JS03	7	2.330	1.163	0.446	0.571	0.571	0.219	1.576	0.564
JS04	4	2.524	1.014	0.546	0.604	0.604	0.096	1.742	0.598
JS05	7	3.173	1.249	0.581	0.685	0.686	0.151	1.348	0.681
JS06	5	2.106	0.817	0.371	0.525	0.526	0.293	0.965	0.522
JS07	6	3.077	1.245	0.597	0.675	0.676	0.115	1.603	0.675
JS09	6	3.596	1.430	0.697	0.722	0.723	0.035	1.949	0.719
JS12	11	3.902	1.558	0.614	0.744	0.744	0.175	1.783	0.731
JS13	7	2.987	1.255	0.581	0.665	0.666	0.126	1.491	0.663
JS14	7	1.174	0.376	0.115	0.148	0.149	0.222	2.683	0.145
JS15	6	2.990	1.207	0.528	0.666	0.666	0.206	1.487	0.663
JS22	6	2.058	1.018	0.432	0.514	0.515	0.159	1.364	0.504
JS28	4	2.501	0.998	0.536	0.600	0.601	0.106	1.330	0.604
BFU-Jr277	7	1.310	0.520	0.108	0.237	0.237	0.545	0.291	0.236
BFU-Jr38	9	3.964	1.598	0.656	0.748	0.748	0.123	1.588	0.507
CUJRD102	5	1.517	0.564	0.312	0.341	0.341	0.085	3.328	0.334
CUJRD462	8	3.915	1.516	0.620	0.745	0.745	0.168	1.72	0.743
JM5446	3	1.024	0.072	0.012	0.023	0.023	0.496	2.900	0.022
SSR18	7	3.436	1.487	0.558	0.709	0.710	0.213	0.875	0.708
ZMZ7	5	1.910	0.876	0.402	0.476	0.477	0.156	1.572	0.486
Mean	6.129	2.379	0.976	0.437	0.511	0.512	0.164	1.649	0.503

*N*_*A*_, Number of alleles; *N*_E_, Effective number of alleles; *I*, Shannon’s information index; *H*_O_, Observed heterozygosity; *H*_E_, Expected heterozygosity; *uH*_E_, Unbiased expected heterozygosity; *F*, Fixation index; *N*_m_, Gene flow; *PIC*, Polymorphic information content

The mean Shannon’s Information index (*I*) was 0.976 and ranged from 0.072 (JM5446) to 1.598 (BFU-Jr38) across loci. In addition, unbiased expected heterozygosity (*uH*_E_) of individual loci ranged from 0.023 at JM5446 to 0.748 at BFU-Jr38 and averaged at 0.512 alleles per locus. The Fixation index (*F*) ranged from 0.020 at JR05 to 0.545 at BFU-Jr277 and averaged 0.164 (Table 1). Moreover, Polymorphic information content (*PIC*) varied from 0.022 (in JM5446) to 0.743 (in CUJRD462), with a mean of 0.503.

## Population genetic diversity and gene flow

The genetic diversity parameters at the population level were considerably different across populations of *J. regia* (Table 2). The number of observed alleles (*N*_A_) averaged 3.141 and varied from 2.323 (GMR) to 3.419 (TPR and GDR). The mean number of effective alleles (*N*_E_) across 27 populations was 2.067, ranging from 1.789 (GMR) to 2.284 (AKR). The observed heterozygosity (*H*_O_) and expected heterozygosity (*H*_E_) across all populations varied from 0.347 (ZAR) to 0.522 (AKR) and 0.371 (SMR) to 0.495 (RGR), with an average of 0.438 and 0.437, respectively.

**Table 2:** Genetic diversity within 27 populations of *Juglans regia* based on SSR data

Code	*N*	*N*_A_	*N*_E_	*I*	*H*_O_	*H*_E_	*uH*_E_	*F*	*A*_R_	*PPL*
ARR	20	3.161	2.036	0.754	0.355	0.419	0.429	0.170	2.342	87.10%
ESR	20	3.129	1.983	0.741	0.405	0.409	0.420	0.033	2.326	87.10%
ETR	20	3.000	1.980	0.706	0.419	0.406	0.416	-0.025	2.220	87.10%
GDR	20	3.419	2.211	0.852	0.515	0.484	0.496	-0.068	2.483	93.55%
KNR	20	3.161	2.024	0.775	0.420	0.441	0.452	0.105	2.358	93.55%
QAR	20	3.194	2.067	0.785	0.464	0.446	0.458	-0.014	2.372	93.55%
RGR	20	3.258	2.264	0.877	0.500	0.495	0.508	-0.020	2.565	93.55%
RUR	20	3.000	2.031	0.734	0.411	0.416	0.426	0.000	2.298	87.10%
SAR	20	3.226	2.105	0.797	0.465	0.450	0.462	-0.008	2.397	93.55%
TER	20	2.935	1.993	0.725	0.391	0.410	0.421	0.028	2.285	90.32%
WER	17	3.258	2.127	0.809	0.386	0.452	0.466	0.110	2.445	93.55%
ZAR	20	3.129	1.931	0.701	0.347	0.388	0.398	0.075	2.241	90.32%
AKR	17	3.290	2.284	0.864	0.522	0.492	0.507	-0.056	2.527	90.32%
EHR	17	3.226	2.211	0.838	0.464	0.481	0.496	0.024	2.468	100.00%
GSR	20	3.194	1.983	0.762	0.459	0.435	0.446	-0.057	2.321	93.55%
HMR	20	3.355	2.231	0.842	0.462	0.467	0.479	-0.007	2.501	90.32%
JBR	19	3.323	2.184	0.841	0.450	0.468	0.481	0.040	2.507	93.55%
KDR	20	3.097	1.917	0.703	0.381	0.393	0.403	0.034	2.241	93.55%
KHR	20	3.387	2.213	0.839	0.494	0.469	0.481	-0.042	2.484	93.55%
KSR	19	3.032	2.018	0.729	0.417	0.412	0.423	0.015	2.288	90.32%
MZR	19	3.226	2.054	0.779	0.475	0.436	0.448	-0.078	2.381	90.32%
NGR	19	3.226	2.209	0.842	0.484	0.482	0.495	-0.015	2.481	90.32%
TAR	21	3.387	2.103	0.812	0.472	0.459	0.471	-0.031	2.408	93.55%
TKR	15	2.387	1.856	0.622	0.428	0.381	0.394	-0.113	2.053	80.65%
TPR	18	3.419	2.183	0.848	0.433	0.472	0.486	0.089	2.512	96.77%
SMR	20	3.065	1.826	0.674	0.348	0.371	0.381	0.119	2.201	96.77%
GMR	7	2.323	1.789	0.602	0.456	0.372	0.404	-0.201	2.040	83.87%
Mean	19	3.141	2.067	0.772	0.438	0.437	0.450	0.005	2.361	91.4%

*N*_A_, Number of alleles; *N*_E_, Effective number of alleles; *I*, Shannon’s information index; *H*_O_, Observed heterozygosity; *H*_E_, Expected heterozygosity; *uH*_E_, Unbiased expected heterozygosity; *F*, Fixation index; *A*_R_, Allelic richness; *PPL*, Percentage of polymorphic loci

The populations AKR (*N*_E_ = 2.284, *H*_O_ = 0.522, *H*_E_ = 0.492 and *I* = 0.864), GDR (*N*_E_ = 2.211, *H*_O_ = 0.515, *H*_E_ = 0.484 and *I* = 0.852), and RGR (*N*_E_ = 2.264, *H*_O_ = 0.5, *H*_E_ = 0.495 and *I* = 0.877) showed high levels of genetic diversity, while those with low values were observed in SMR (*N*_E_ = 1.826, *H*_O_ = 0.348, *H*_E_ = 0.371 and *I* = 0.674) and GMR (*N*_E_ = 1.789, *H*_O_ = 0.456, *H*_E_ = 0.372 and *I* = 0.602). The fixation index (*F*) averaged 0.005, ranging from -0.201 (GMR) to 0.170 (ARR). The low level of fixation index identifies the deficiency of heterozygosity in the *J. regia* populations. The high values of *uH*_E_ were observed in populations RGR (0.508) and AKR (0.507), whereas the low *uH*_E_ values belong to populations SMR (0.381) and TKR (0.394). *A*_R_ values ranged between 2.040 (GMR) and 2.565 (RGR). As with the case of *uH*_E_, population RGR also represented a high *A*_R_ level. The mean polymorphic loci (*PPL*) percentage across 27 populations was high (91.40%) and ranged from 80.65% for TKR to 100% for EHR (Table 2). The gene flow (*N*m) between populations averaged 1.840. The highest value of *N*m was observed between ARR and TKR populations (0.857), whereas the lowest value belongs to TAR and TPR populations (5.614) (Table S4).

### Population clustering and genetic structure

#### Patterns of genetic differentiation

**Fig. 1:**
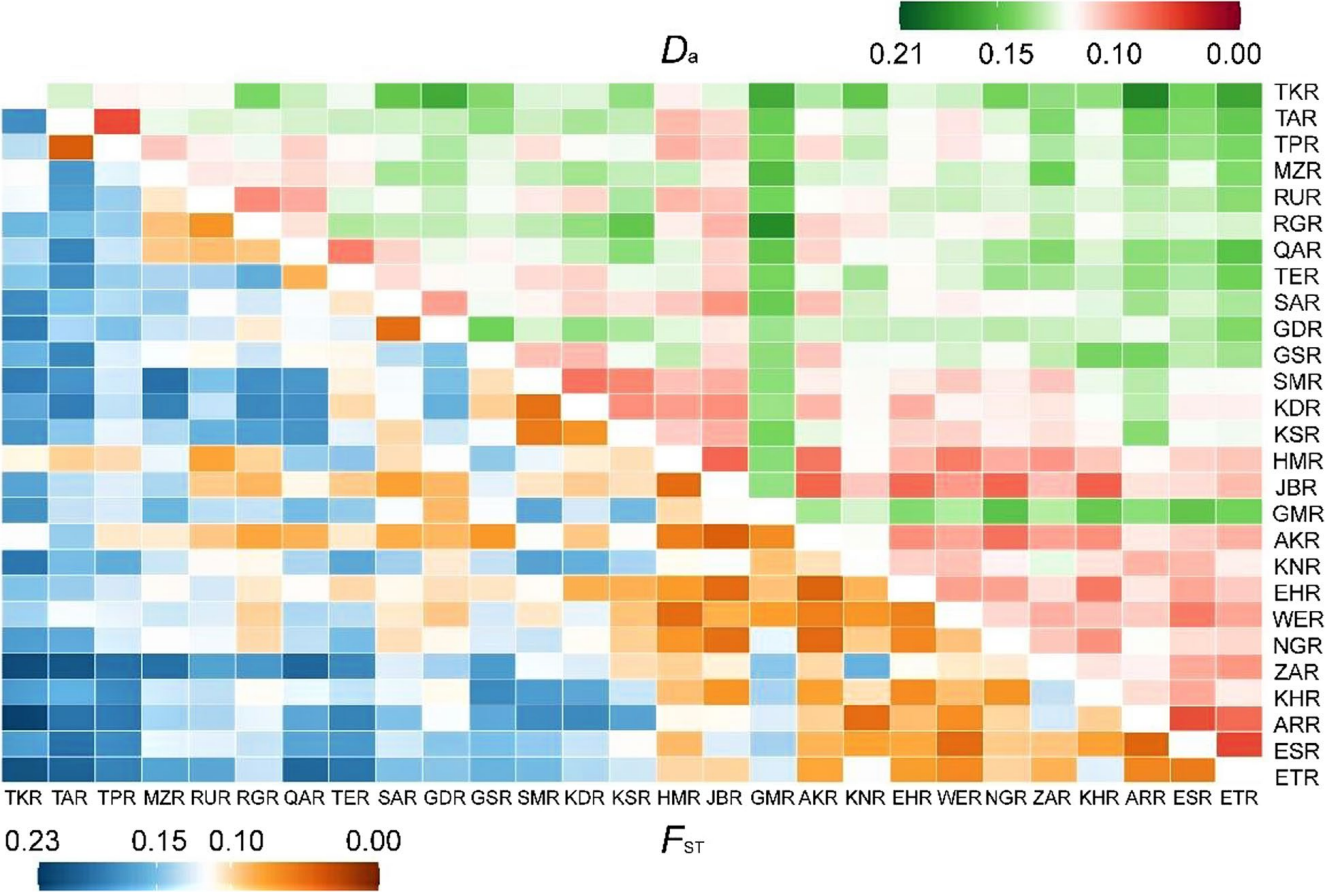
Heat map depicting pairwise genetic distance between populations. The left-lower half represents the genetic differentiation (*F*_ST_); the right-upper half indicates the genetic distance (*D*_A_)

The genetic differentiation coefficient (*F*_ST_) was moderate, ranging from 0.042 between TPR and TAR to 0.225 between ARR and TKR. Moreover, pairwise comparative analysis of Nei’s genetic distance values represents minimum range of 0.046 between ETR and ESR to 0.200 between ARR and TKR (Fig. 1, Table S3). The data demonstrated that populations ARR and TKR are genetically far from each other in terms of genetic distance and genetic differentiation. Also, the results of AMOVA analysis indicated that variation within populations was 79%, while variation among populations was 21% (Table 3), possibly because of the high gene flow (*N*_m_ = 1.840) between *J. regia* populations.

**Table 3:** Analysis of molecular variance (AMOVA) of 27 populations of *Juglans regia*

Scale	Source	*d.f*	Sum of squares	Mean squares	Percentage of variation (%)
Total	Among Pops	26	2379.375	91.514	21%
	Within Pops	486	7492.773	15.417	79%
	Total	512	9872.148		100%
	*F*_ST_	0.206			
	*P*	< 0.001			

*d.f*. degree of freedom

#### Two main genetic groups

**Fig. 2:**
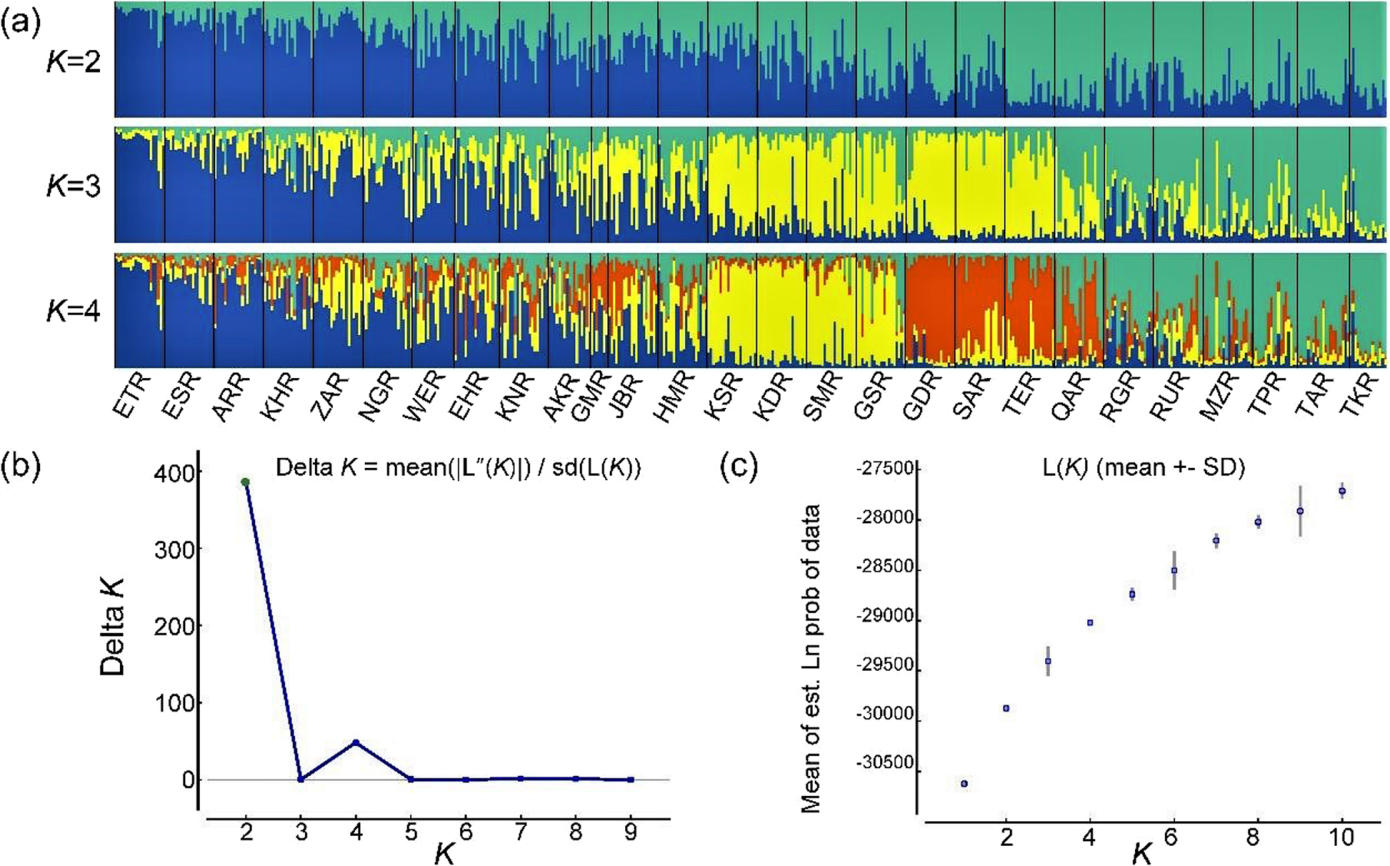
Bayesian inference of the number of clusters (*K*) from 2 to 4 of *J. regia* by STRUCTURE. (**a**) Different colors indicate various genetic components, green for G1, blue for G2, yellow for G3, and orange for G4. (**b**) The optimal *K* value using the Delta *K* (*ΔK*) method. (**c**) Mean log-likelihood of the data at varying estimates of *K*

**Fig. 3:**
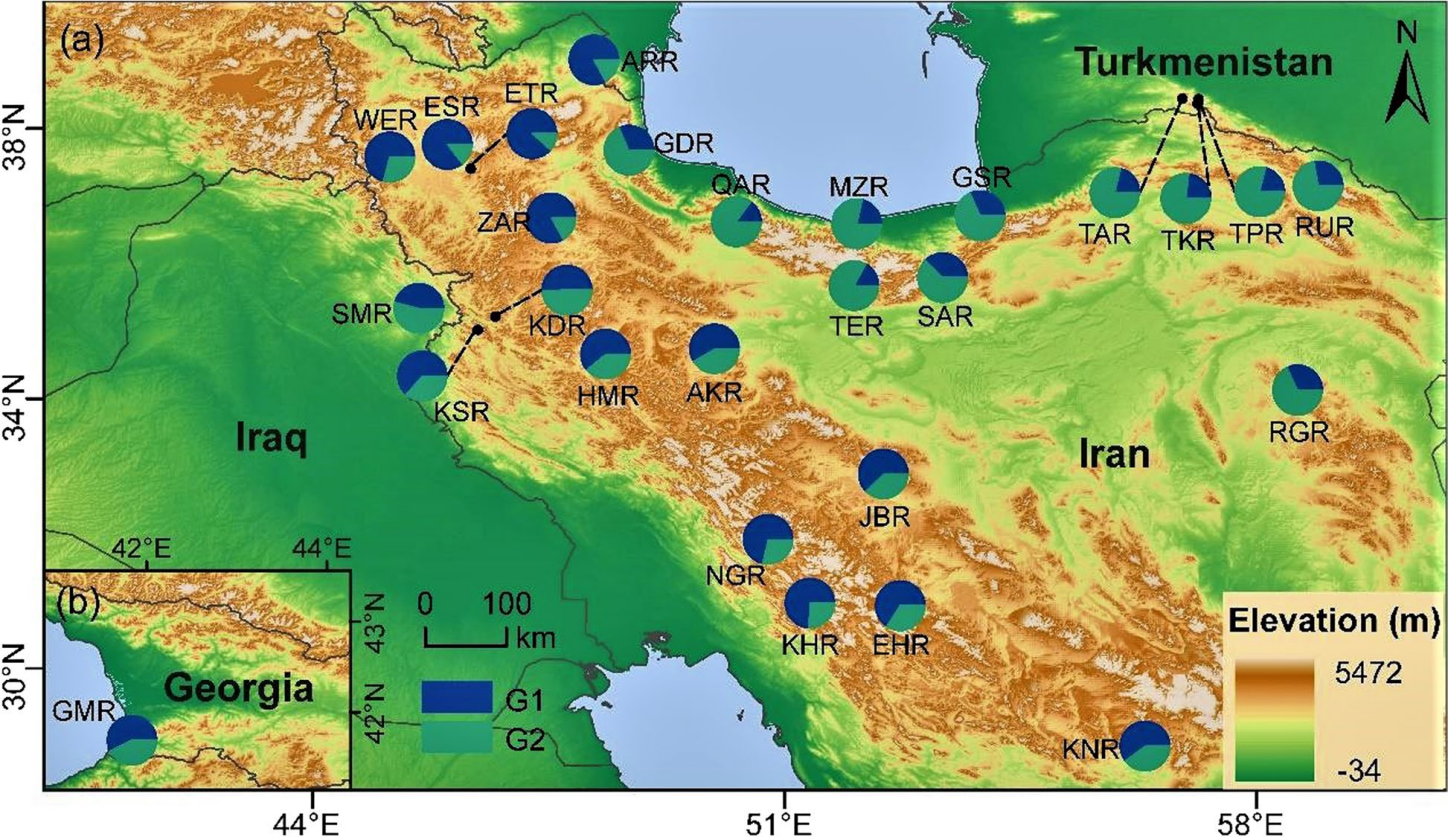
Geographical distribution of the genetic structure in 27 populations across the Iranian Plateau. Right-lower inset represents the population from Georgia. The proportion of the pie charts are based on the STRUCTURE results in Fig. 2a (*K* = 2). The designation of G1 and G2 corresponds to Fig. 2a

**Fig. 4:**
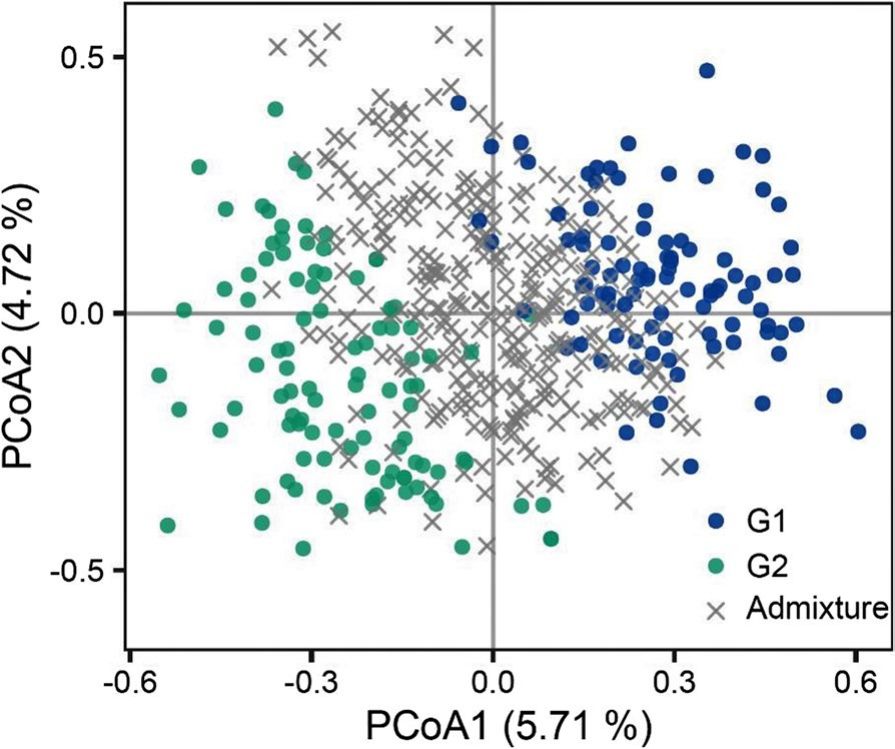
Principal Co-ordinates Analysis (PCoA) of 508 individuals based on 31 microsatellite loci. The designation of G1 and G2 corresponds to Fig. 2a, while the grey crosses indicate mixed individuals

**Fig. 5:**
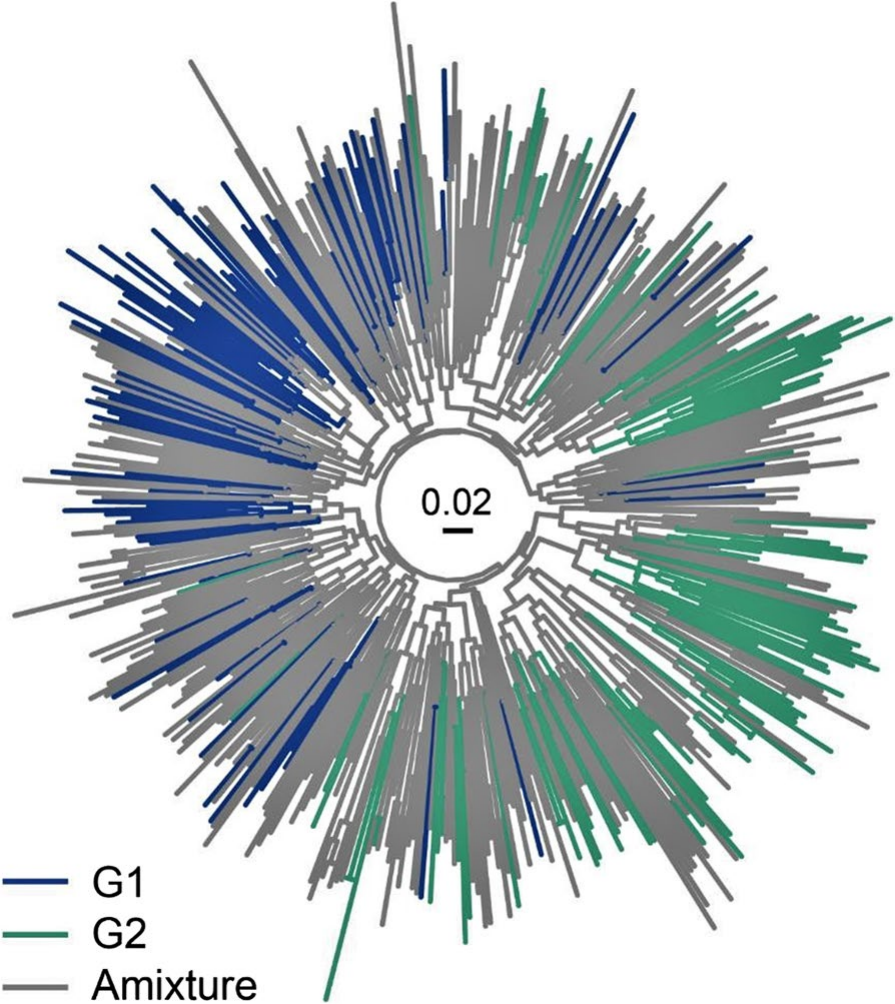
Neighbor-joining tree of 508 individuals, colors correspond to Fig. 4. The designation of G1 and G2 corresponds to Fig. 2a, while the grey branches represent mixed individuals

Bayesian structure analysis of 508 samples revealed the maximum peak at *K* = 2, and one smaller peak at *K* = 4, hence *K* = 2, 3, and 4 are shown (Fig. 2). At *K* = 2, the populations of *J. regia* were grouped into two genetic clusters. The first genetic cluster (green) mainly contained 213 individuals from 11 populations, including GSR, GDR, SAR, TER, QAR, RGR, RUR, MZR, TPR, TAR, and TKR. The second comprises 292 accessions collected from 16 populations (blue, Fig. 2a). Generally, lots of admixtures were detected among most populations, except populations TER, QAR, ARR, ESR, ETR, and ZAR. Populations corresponding to these groups were geographically differentiated. Each population and its proportion are represented as a pie chart in Fig. 3. Consistent with STRUCTURE results, *K* = 2, the populations were separated into two clusters in the PCoA (Fig. 4) and NJ analyses (Figs. 5 & S1). In PCoA, both the first (5.71%) and second (4.72%) axes generally separated individuals of *J. regia* into two main groups (blue and green ones). The admixtures were grouped between the above groups (Fig. 4).

In addition, at the maximum likelihood clustering with *K* = 3, the number of optimum groups was three. Cluster 1 consisted of six TKR, TAR, TPR, MZR, RUR, and RGR (green) populations. Cluster 2 arises from 13 populations, including HMR, JBR, GMR, AKR, EHR, KNR, WER, NGR, ZAR, KHR, ARR, ESR, and ETR (blue). Cluster 3 contained eight populations in QAR, TER, SAR, GDR, GSR, SMR, KDR, and KSR (yellow). These results were consistent with the results from the PCoA (Fig. S2). Further, 508 individuals were assigned to four clusters at *K* = 4. Populations from Turkmenistan, the northeast, and one population from north of Iran (TKR, TAR, TPR, RUR, RGR, and MZR) were grouped in cluster 1 (green). While cluster 2 (blue) encompassed the populations of HMR, JBR, GMR, AKR, EHR, KNR, WER, NGR, ZAR, KHR, ARR, ESR, and ETR. Accessions in cluster 3 were mainly from the west of Iran (yellow), with the GSR population being an exception, not following this pattern. Populations from the Center and north of Iran, including QAR, TER, SAR, and GDR, were grouped in cluster 4 (orange) (Fig. 2a). Each population and its proportion are represented in figure S3 according to colours depicting the genetic cluster identified in STRUCTURE. The STRUCTURE analysis showed gene penetration among the 508 individuals in the four clusters (Figs. S4 & S5).

#### Mantel Test

Simple and partial Mantel tests were carried out to explore the correlations between genetic distance (*F*_ST_) and environmental factors. The results stipulated a weak correlation between genetic distance and geographical distance (*r* = 0.26, *P* = 0.05; Fig. S6a) and between genetic diversity and altitude (*r *= -0.19, *P* = 0.05; Fig. S6b) in the whole dataset.

## Discussion

### Genetic diversity of common walnut populations

Our findings indicate that populations of *J. regia* from the Iranian Plateau possess moderate genetic diversity. The average *H*_O_ and *H*_E_, and *N*_E_ in the current study were lower than in the previous studies [9, 42-46] (Table 4). The lowest genetic diversity was observed in the populations from Iraq and Georgia. The low genetic diversity in the latter might be attributed to a small sample size. The presence of low genetic diversity in SAM population indicates that the main factors behind this might be ecological factors. The population from Iraq, which is situated in a relatively low-altitude area (1186 m), will be most affected by human activities and global warming. Plenty of alleles will be lost due to overexploitation and inappropriate climate warming, thus genetic diversity will be considerably reduced. Additionally, habitat changes and human interference impact the genetic diversity of the SMR population in some ways. The SMR population is located in tourist hotspot, where the natural habitat was disturbed, which may directly cause their genetic variation decrease.

**Table 4:** Comparison of genetic diversity of *Juglans regia* between current study and previous ones which used microsatellite markers

	**Current study**	Karimi et al. (2010) [9]	Karimi et al. (2014) [43]	Vahdati et al. (2014) [45]	Vahdati et al. (2015) [44]	Ebrahimi et al. (2016) [42]	Magige et al. (2022) [46]
*H*_O_	**0.438**	0.682	0.659	0.230	0.659	0.620	0.563
*H*_E_	**0.437**	0.666	0.657	0.720	0.657	0.670	0.558
*N*_E_	**2.067**	2.700	3.040	5.160	3.040	3.240	-

Populations of RGR, AKR, and GDR possessed the highest genetic diversity. According to our observations in the field and enquiries from the farmers, some samples were almost 1000 years old in the AKR population. In addition, the population GDR has been considered a wild stand in Iran [47, 48]. Therefore, the remaining trees in Talesh (GDR) and populations from Arak (AKR) imply the reservation of an invaluable genetic source. We presumed that heterogeneous environment of habitat and climatic features might drive the local adaptation, preserving the genetic variation of RGR, AKR, and GDR populations at the same time. GDR population is located in remote areas, which is advantageous to maintaining high genetic diversity. However, we did not conduct a habitat survey, so, the factors that led to the high level of genetic differentiation of these populations should be elucidated in future studies.

### Population genetic structure among *J. regia* populations

The gene flow and genetic differentiation coefficient are critical parameters for realizing the population structure [49]. The genetic differentiation coefficient (*F*_ST_) varied from 0.042 to 0.225, with an average of 0.124 within 508 *J*. *regia* individuals (Table S3). According to Wright’s classification [50], the *F*_ST_ value indicates moderate differentiation among the populations. Different factors might cause moderate population differentiation, such as pollen and seed diffusion, geographic isolation, breeding system, and environmental heterogeneity [51]. The present study’s most feasible explanation for moderate genetic differentiation could be the out-crossing and wind-pollination of *J. regia*. The close genetic distances between TPR/TAR accessions (0.042) and AKR/JBR (0.044) showed that these populations share many common alleles and are closely related. The adjacency might justify the genetic affinity between these populations.

The results of the AMOVA analysis indicated that 21% of the variation belonged to the differences among populations (*P* < 0.001), and 79% was attributed to the differences within populations. Significant variation within populations and a small variance between populations could be a result of the wind pollination system [52, 53], or a broad exchange of seeds among farmers from different regions. Hence, when selecting populations with high genetic diversity for breeding programs, the emphasis should be on individuals within the population.

The mean gene flow (*N*_m_) was 1.84; if *N*_m_ is = 1, which indicates high-intensity gene flow between populations [50], preventing genetic drift and decreasing the genetic differentiation among populations [54]. Our results suggest that gene flow is one of the main factors influencing the genetic structure of *J. regia* populations. Therefore, the differentiation within populations was remarkably greater than between populations. In contrast to previous studies [44, 55], our findings demonstrated that ecological and geological features such as Zagros, Alborz, and Kopet Dagh mountains and deserts were not a barrier to gene flow as previously envisioned. Therefore, we postulated that the current level of moderate diversity is due to sustaining a high level of gene flow in this species in Iran and neighboring countries. Pollen spreading is probably the determinant mechanism of gene flow among populations [45]. Besides the natural factors, human-mediated selection, and exchanges of the germplasms, such as choosing fat-rich nuts, could have likely contributed to the distribution and level of genetic differentiation of *J. regia*.

The three complementary approaches, NJ, PCoA, and STRUCTURE, employed to investigate the structure of the *J. regia* accessions mostly confirmed each other. The findings reflected consistency with the accession’s geographic distribution pattern.

Iraq, Georgia, and Turkmenistan are geographically located in the west, northwest, and northeast of Iran, respectively. The Kopet Dagh mountain range is on the border between Turkmenistan and Iran [56]. Moreover, Hawraman or Uramanat is a mountainous region divided between the provinces of Kermanshah and Kurdistan (west of Iran), and the northeast of the Kurdistan Region in Iraq. The STRUCTURE and NJ analyses placed them adjacent to their neighboring populations in Iran. These data prove that walnut populations in Georgia, Turkmenistan, and Iraq may have originated from the same historic gene pools as their counterparts in Iran. In addition, Georgia does not have a common border with Iran, but is located near the northwestern populations of Iran. The finding was inferred to indicate that human activities may have promoted long-distance dispersal. Samples from Georgia have also been assembled in one cluster with Iranians’ in Pollegioni et al. [17]. Additionally, a lot of admixtures were detected among all populations that might reflect allele sharing [57].

The relationship between molecular data of *J. regia* populations and environmental factors was investigated using the simple and partial Mantel tests. The findings indicated a weak correlation between the genetic and geographic distance among the populations (*r* = 0.26, *P* = 0.05). The genetic structure of some populations in the plateau was not influenced by geographic distribution; for example, grouping accessions from the different regions in a similar cluster (e.g., GSR) could result from low genetic variation among the populations suggesting that the genetic structure of *J. regia* populations does not always correspond to their geographical regions. The reduced genetic structure, even in broad geographic barriers including the Lut desert, Alborz and Zagros Mountain ranges, can be attributed to human-mediated gene flow among the populations. Further investigation to identify the determinant reasons is needed.

The partial Mantel test also exhibited a weak correlation between the genetic distance and altitude (*r* = -0.19, *P* = 0.029). It could be speculated that the genetic differentiation of *J. regia* populations in the Iranian Plateau might be affected by altitude. Nevertheless, further research using additional samples from the neighboring countries such as Afghanistan and Pakistan, and other molecular markers (e.g., SNP) will be helpful to provide a more accurate conclusion on the driving force behind the population structure in the Iranian Plateau. In addition, considering the presence of many commercial walnut cultivars around the world, it is suggested to include commercial cultivars with comrephesive sampling scheme in future research for a better population genetic comparison between native populations and native plantation.

### Conservation implications

A comprehensive insight into the genetic differentiation and structure are prerequisites to devising species preservation measures [58]. From a conservation standpoint, the maximum genetic differentiation was identified within AKR, RGR, and GDR populations. The GDR population has been considered a wild stand in Iran [47, 48]. As mentioned earlier, the AKR population includes some trees that are more than 1000 years old. These characteristics are encouraging for preserving the genetic resources of these populations for in situ conservation. There is an urgent need to increase interpopulation genetic diversity for SMR populations that showed low genetic diversity and approaches through assistant migration whereby methods such as such as propagation, seedling management, and tissue culture could be the most effective.

## Conclusions

Our analyses provide the most comprehensive investigation, to date, on the genetic diversity and population structure of *J. regia* in the Iranian Plateau. The findings revealed moderate genetic differentiation and high gene flow, which were attributed to its out-crossing mating system and anthropogenic activities. In addition, the data generated here confirmed that the accessions contained a relatively high level of genetic variation and a weak correlation between the genetic and geographic distance of *J. regia*. The common walnut populations of the Iranian Plateau can be divided into two main genetic groups, but with a wide genetic exchange. Moreover, our results provide insights into incorporating the most diverse populations, including AKR, RGR, and GDR into germplasm resources conservation. Lastly, we put forward an extended sampling of *J. regia* populations from more countries to enhance better understanding the genetic relationship of *J. regia* in the Iranian Plateau with other regions.

## Methods

### Sampling

**Fig. 6:**
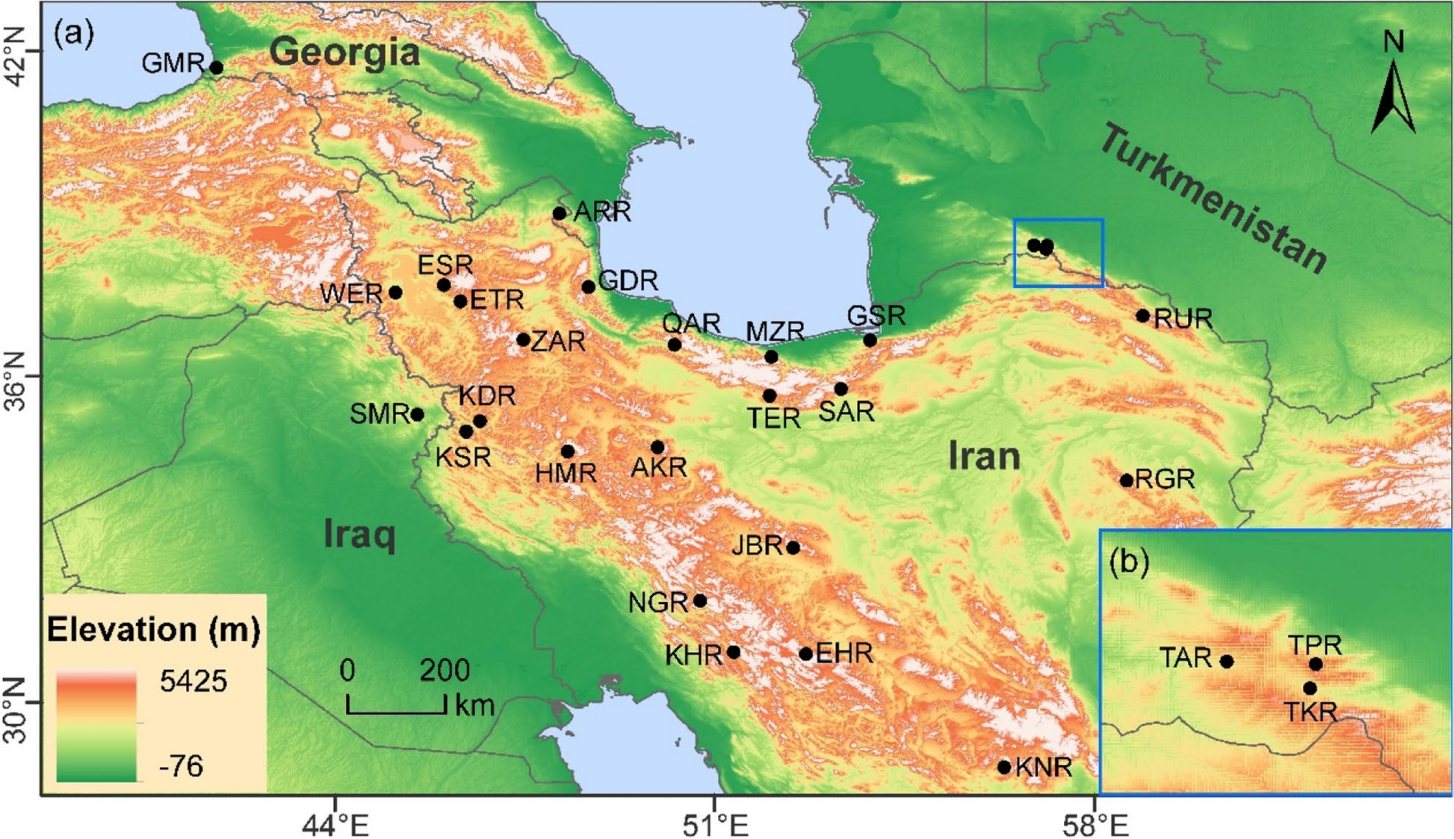
Geographical distribution of the 27* J*. *regia* populations (see Table S1 for detailed sampling information). (**a**) The geographical location of the populations; (**b**) The right-lower inset shows the geographical location of three populations from Turkmenistan

A total of 508 walnut trees from 27 populations were collected from 21 provinces of Iran and one location each from Georgia and Iraq, and three from Turkmenistan with various climates and altitudes (Fig. 6) during the spring and summer of 2019.

We planned to collect ca. 20 trees per location, but 11 populations had sample sizes ranging from 7 to 21 individuals. The geographical location of each population was recorded (Table S1). Depending on their availability to avoid consanguinity, the trees were at least 100 m apart, with a trunk diameter of more than 100 cm, and at least 100 years old based on interviews with orchard owners and local people. The human participants (orchard owners and local people) who were interviewed are not directly involved in this study. All healthy leaf samples intended for DNA extraction were collected and dried. Voucher specimens were deposited at the herbarium of Yasouj University Herbarium (YUH) in Iran.

### DNA extraction and PCR amplification

Total DNA was extracted from ca. 20 mg of dry leaves according to a modified CTAB method [59, 60]. DNA concentration and quality were measured on 1% TAE agarose gels and using a NanoDrop®ND-1000 spectrophotometer (Thermo Fisher Scientific, Wilmington, DE, USA); subsequently, all samples were diluted to 30–50 ng/mL for PCR reactions. Previous studies [46, 61] described a multiplex of 31 pairs was used for genotyping the 508 walnut trees (Tables 1, S2). PCR amplification and cycling conditions were performed according to Magige et al. [46]. Briefly, pre-denaturation at 98 °C for 2 min, 35 cycles of denaturation at 98 °C for 10 s, primer annealing at 53–61 °C for 15 s, extension at 72 °C for 10 s, and a final extension at 72 °C for 5 min, with a holding temperature of 4 °C. The fragment sizes of PCR products were separated with an ABI 3730xl automated sequencer (Applied Biosystems, Foster City, CA, USA). GENEMARKER v4.0 (SoftGenetics, State College, PA, USA) was applied to score the SSR data as diploid genotypes.

### Statistical analysis

To assign the level of the genetic diversity of loci and populations, genetic diversity analysis, such as number of alleles (*N*_A_), observed heterozygosity (*H*_O_) and expected heterozygosity (*H*_E_), genetic differentiation coefficient (*F*_ST_), gene flow (*N*_m_), fixation index (*F*_IT_), polymorphic information content (*PIC*), inbreeding coefficient (*F*_IS_), unbiased expected heterozygosity (*uH*_E_) across loci, Nei’s genetic distances between populations, and analysis of molecular variance (AMOVA) were performed in GeneAlex v.6.5 [62]. Allelic richness (*A*_R_) was assigned with the R package “hierfstat”(https://cran.r-project.org/web/packages/hierfstat) package v.3.0.7 [63] for R version 3.6.3 [64]. Assessing of the pairwise genetic differentiation (*F*_ST_) and Nei’s genetic distance (*D*_A_) between pairs of populations was performed using Arlequin v.3.5 [65] and MSA v.4.05 [66]. Then, the data was reflected on a heatmap by the R package “ggplot” [67].

The genetic assignment of each individual was implemented in STRUCTURE v.2.3.4 [68]. The run parameters were set as follows: burn-in period of 100,000 iterations, length of 1,200,000 Markov Chain Monte Carlo (MCMC) generations to increase *K* values from 1–10. Each *K* was repeated in 20 simulations. The optimum value of *K* was evaluated according to the Delta *K* criterion using STRUCTURE HARVESTER v.0.6.1 [69], and repeated sampling analysis of the results was carried out in CLUMMP v.1.1.2 [70]. Finally, the Distruct v.1.1 software [71] was used to map the results, and STRUCTURE graphical results were plotted with Distruct. The populations were represented on the topographical map according to their relative proportions to the genetic clusters generated from STRUCTURE using ArcGIS v.10.7 (ESRI, Redlands, CA, USA) [72]. The value of *Q* estimated the affiliation probabilities of each genotype in every cluster, and genotypes were assigned to their relevant clusters based on a threshold value of 0.80. According to Wambulwa et al. [34], individuals with a high percentage of membership (*Q* = 0.80) in any of the genetic clusters were defined as distinct genetic groups, and the individual with low probabilities (*Q* < 0.80) were treated as “admixture”.

Moreover, structure in the distribution of genetic differentiation was plotted by principal coordinates analysis (PCoA) using Nei’s genetic distance in R package “ggplot2” [73]. In addition, a graphical presentation of the genetic structure of *J. regia* populations was acquired by applying the neighbor-joining (NJ) method with 1000 bootstrap replicates in Populations v.1.2.31 [74]. R package “ggtree” [67] was used to visualize the result. Further, the simple and partial Mantel tests were performed with the “vegan” package v.2.5–3 [75] for R version 3.6.3 [64] for correlation between *F*_ST_ genetic distance, altitude, and geographic (km) differences for the dataset of *J. regia*. Significance was evaluated by conducting 1000 permutations.

## Supplementary Information

**Additional file 1:** **Table S1.** Detailed sampling information of 27 *Juglans regia* populations across the Iranian Plateau. **Table S2.** Detailed information for the 31 pairs of primers and their combination for multiplex PCR. **Table S3.** Population pairwise *F*_ST_ and Nei’s genetic distance (*D*_A_). Lower-left part: *F*_ST_; upper-right part: *D*_A_. **Table S4.** Gene flow (*N*_m_) among populations. **Fig. S1. **Neighbor-joining tree of 508 individuals, colors correspond to Fig. 4. The designation of G1 and G2 corresponds to Fig. 2a, while the grey branches represent mixed individuals. **Fig. S2. **Principal Co-ordinates Analysis (PCoA) analysis of 508 individuals based on Nei’s genetic distance (*D*_A_). The groups were defined according to STRUCTURE analysis (*K*=3, *Q*> 0.8). **Fig. S3.** Geographical distribution of the genetic structure of 27 populations. Pie charts show the genetic proposition of each cluster in STRUCTURE analysis (*K*=4). **Fig. S4.** Principal Co-ordinates Analysis (PCoA) of 508 individuals based on Nei’s genetic distance (*D*_A_). The groups were defined according to STRUCTURE analysis (*K*=4, *Q*> 0.8). **Fig. S5. **Neighbor-joining tree of 508 individuals. Colors correspond to Fig. S4. **Fig. S6.** Genetic isolation by distance of 27 populations in the Iranian Plateau. (a) genetic distance and geographical distance (*r*= 0.26, *P*= 0.05), (b) genetic distance and altitude (*r*= -0.19, *P*= 0.05).

## Data Availability

All data from this study are incorporated in this article and its supplementary information.

## Abbreviations

*N*_A_: Number of alleles
*N*_E_: Effective number of alleles
*I*: Shannon’s information index
*A*_R_: Allelic richness
*PPL*: Percentage of polymorphic loci
*PIC*: Polymorphic information content
*H*_O_: Observed heterozygosity
*H*_E_: Expected heterozygosity
*F*_ST_: Genetic differentiation coefficient
*N*_m_: Gene flow
*F*_IT_: Fixation index
*F*_IS_: Inbreeding coefficient
*uH*_E_: Unbiased expected heterozygosity
d.f.: Degree of freedom
*D*_A_: Nei’s genetic distance
SSR: Simple Sequence Repeats
PCoA: Principal coordinate analysis
NJ: Neighbor-joining
AMOVA: Analysis of molecular variance
IR: Iran
TM: Turkmenistan
IQ: Iraq
GE: Georgia
