# Supplementary material for: Unravelling the genetic diversity and population structure of common walnut in the Iranian Plateau
Source: BMC Plant Biol. 2023 Apr 18;23:201. doi: 10.1186/s12870-023-04190-2 (PMC10111805; doi:10.1186/s12870-023-04190-2)
Supplement: Supplementary file 1 — Additional file 1: Table S1. Detailed sampling information of 27 Juglans regia populations across the Iranian Plateau. Table S2. Detailed information for the 31 pairs of primers and their combination for multiplex PCR. Table S3. Population pairwise FST and Nei’s genetic distance (DA). Lower-left part: FST; upper-right part: DA. Table S4. Gene flow (Nm) among populations. Fig. S1. Neighbor-joining tree of 508 individuals, colors correspond to Fig. 4. The designation of G1 and G2 corresponds to Fig. 2a, while the grey branches represent mixed individuals. Fig. S2. Principal Co-ordinates Analysis (PCoA) analysis of 508 individuals based on Nei’s genetic distance (DA). The groups were defined according to STRUCTURE analysis (K=3, Q> 0.8). Fig. S3. Geographical distribution of the genetic structure of 27 populations. Pie charts show the genetic proposition of each cluster in STRUCTURE analysis (K=4). Fig. S4. Principal Co-ordinates Analysis (PCoA) of 508 individuals based on Nei’s genetic distance (DA). The groups were defined according to STRUCTURE analysis (K=4, Q> 0.8). Fig. S5. Neighbor-joining tree of 508 individuals. Colors correspond to Fig. S4. Fig. S6. Genetic isolation by distance of 27 populations in the Iranian Plateau. (a) genetic distance and geographical distance (r= 0.26, P= 0.05), (b) genetic distance and altitude (r= -0.19, P= 0.05). [file 12870_2023_4190_MOESM1_ESM.docx]

**BMC Plant Biology**

Supplementary Materials for

**Unravelling the genetic diversity and population structure of Persian walnut in the Iranian Plateau**

Robabeh Shahi Shavvon^1*^, Hai-Ling Qi^2,3,4^, Mohammad Mafakheri^5^, Pen-Zheng Fan^3,6^, Hong-Yu Wu^2,6^, Fatemeh Bazdid Vahdati^7^, Hanady S. Al-Shmgani^8^, Yue-Hua Wang^4^, Jie Liu^2,3^*

*Correspondence: [r.shahi@yu.ac.ir](mailto:r.shahi@yu.ac.ir), liujie@mail.kib.ac.cn

^1^Department of Biology, Faculty of Science, Yasouj University, Yasouj, Iran

^2^CAS Key Laboratory for Plant Diversity and Biogeography of East Asia, Kunming Institute of Botany, Chinese Academy of Sciences, Kunming, Yunnan 650201, China

^3^Germplasm of Bank of Wild Species, Kunming Institute of Botany, Chinese Academy of Sciences, Kunming, Yunnan 650201, China

^4^School of Ecology and Environmental Science, Yunnan University, Kunming 650091, China

^5^Department of Plant Sciences, University of California - Davis, Davis, CA 95616, USA

^6^University of Chinese Academy of Sciences, Beijing 100049, China

^7^Department of Biology, Faculty of Science, University of Guilan, Rasht, Iran

^8^Department of Biology, College of Education for Pure Sciences (Ibn Al-Haitham), University of Baghdad, Baghdad, Iraq

**This file includes:**

Tables S1, S2, S3, and S4.

Figs. S1 to S6

| **Table S1.** Detailed sampling information of 27 *Juglans regia* populations across the Iranian Plateau. | | | | | |
| --- | --- | --- | --- | --- | --- |
| Code | Locality | Latitude | Longitude | Altitude | Sample Size |
| AKR | Tafresh, Arak, IR | 34.709 | 49.949 | 2001 | 17 |
| ARR | Ardabil, Ardabil, IR | 39.003 | 48.146 | 1071 | 20 |
| EHR | Eghlid, Fars, IR | 30.898 | 52.686 | 2219 | 17 |
| ESR | Azarshahr, East Azerbaijan, IR | 37.683 | 46.011 | 1897 | 20 |
| ETR | Maragheh, East Azerbaijan, IR | 37.387 | 46.323 | 1677 | 20 |
| GDR | Talesh, Guilan, IR | 37.651 | 48.683 | 1124 | 20 |
| GMR | Mtirala National Park, Ghoma mountain, GE | 41.686 | 41.833 | 830 | 7 |
| GSR | Bandare-Gaz, Golestan, IR | 36.671 | 53.864 | 480 | 20 |
| HMR | Toyserkan, Hamadan, IR | 34.636 | 48.292 | 1794 | 20 |
| JBR | Kuhpayeh, Isfahan, IR | 32.854 | 52.447 | 2337 | 19 |
| KDR | Uraman Takht, Kurdestan, IR | 35.188 | 46.684 | 2011 | 20 |
| KHR | Dena, Kohgiluyeh and Boyer-Ahmad, IR | 30.936 | 51.350 | 1970 | 20 |
| KNR | Bam, Kerman, IR | 28.818 | 56.330 | 2021 | 20 |
| KSR | Kermanshah, Kermanshah, IR | 34.993 | 46.423 | 1742 | 19 |
| MZR | Nur, Mazandaran, IR | 36.366 | 52.043 | 615 | 19 |
| NGR | Naghan, Chaharmahal and Bakhtiari, IR | 31.883 | 50.731 | 1869 | 19 |
| QAR | Qazvin, Qazvin, IR | 36.592 | 50.264 | 1737 | 20 |
| RGR | Gonabad, Razavi Khorasan, IR | 34.094 | 58.588 | 1969 | 20 |
| RUR | Quchan, Razavi Khorasan, IR | 37.123 | 58.890 | 1700 | 20 |
| SAR | Mahdishahr, Semnan, IR | 35.773 | 53.328 | 1992 | 20 |
| SMR | Zalam, Ahmad awa, Barkala, Sulaymaniyah, IQ | 35.298 | 45.526 | 1186 | 20 |
| TAR | Upper Aidere, TM | 38.421 | 56.882 | 1151 | 21 |
| TER | Damavand, Tehran, IR | 35.651 | 52.014 | 1802 | 20 |
| TKR | Ipaikala, TM | 38.350 | 57.104 | 1055 | 15 |
| TPR | Karayalchi, TM | 38.414 | 57.119 | 1349 | 18 |
| WER | Urmia, West Azerbaijan, IR | 37.552 | 45.121 | 1349 | 17 |
| ZAR | Mahneshan, Zanjan, IR | 36.679 | 47.481 | 1530 | 20 |
| Notes: IR, Iran; TM, Turkmenistan; IQ, Iraq; GE, Georgia. | | | | | |

| **Table S2.** Detailed information for the 31 pairs of primers and their combination for multiplex PCR. | | | | | |  |  |
| --- | --- | --- | --- | --- | --- | --- | --- |
| Locus | Primer pair sequence (5'-3') | Repeat motif | Size range (bp) | Ta (℃) | Reference | Dye | Multiplexing information |
| JR02 | F:GTTGGGCTGCCAGAGATTCT | (TTC)7 | 141-168 | 56 | Unpublished | HEX | M 3 |
|  | R:ACGCTTCATTGGTAAACGAACG |  |  |  |  |  |  |
| JR03 | F:ATACGGATCTGATGGCATGG | (GAC)6 | 235-268 | 56.7 | Unpublished | HEX | M 1 |
|  | R:AGACAGCAATATCCACCCTT |  |  |  |  |  |  |
| JR04 | F:TGTTCTACCATTGCTCCGAA | (TCA)6 | 348-381 | 57 | Unpublished | FAM | M 4 |
|  | R:ACACCTAGTTAGGAGCTGGA |  |  |  |  |  |  |
| JR05 | F:GTCGCAAGCTCAGCAAATAA | (AAAG)8 | 194-214 | 57 | Unpublished | HEX | M 2 |
|  | R:TGTATGTATGGGAGGGGGAT |  |  |  |  |  |  |
| JR06 | F:TTGGAGCCCAATCAAGGATT | (ACAG)5 | 299-319 | 57 | Unpublished | HEX | M 3 |
|  | R:CACACAGAAAAGACCAGCAG |  |  |  |  |  |  |
| JR07 | F:TCTTAAGAAGAGCCAATCGC | (ACCA)5 | 303-330 | 56.1 | Unpublished | FAM | M 2 |
|  | R:GCTGTGTACCTCTTAGGGTT |  |  |  |  |  |  |
| JR08 | F:ACTCCTGTCACTTGTATGCC | (CACG)5 | 329-359 | 57.2 | Unpublished | FAM | M 3 |
|  | R:CCCGAGACATCAGAACCTTT |  |  |  |  |  |  |
| JR09 | F:ATCACCTGATGTGGAAGCAA | (GAGGA)5 | 359-394 | 56.8 | Unpublished | TAMRA | M 5 |
|  | R:CCATAGGACCCATAACGTGA |  |  |  |  |  |  |
| JR10 | F:TGGGAAGGGATTTCGTGTTGT | (TCTGA)5 | 195-215 | 56 | Unpublished | TAMRA | M 4 |
|  | R:TAAGGACGCCCATTGCCATT |  |  |  |  |  |  |
| JR11 | F:AGCTAGCTCTCAAACAACAAGC | (GCAGTA)8 | 140-164 | 53 | Unpublished | FAM | M 2 |
|  | R:ACAAACATGGCAACCTTCGTG |  |  |  |  |  |  |
| JR12 | F:GCCTCTCCTCGTGCTCATTT | (GAA)18 | 212-230 | 56 | Unpublished | TAMRA | M 3 |
|  | R:ACTCGCTACTTTTCAGGCCC |  |  |  |  |  |  |
| JS02 | F:CAACTCTGTGATTGCATGGG | (AAAG)8 | 383-411 | 57 | Xu et al. 2020 | HEX | M 5 |
|  | R:GGTAACTCTCATCGCTAGGG |  |  |  |  |  |  |
| JS03 | F:TGACGAGGTTTACCAGATGGG | (GAA)5 | 90-105 | 58 | Xu et al. 2020 | TAMRA | M 2 |
|  | R:CGTTCTTCTTTCAGAGTGCTGTT |  |  |  |  |  |  |
| JS04 | F:CATACATATGTGGGTGGCCT | (GAA)6 | 349-367 | 56.8 | Xu et al. 2020 | TAMRA | M 2 |
|  | R:TCCTCCTCTCTCTTCCCTTT |  |  |  |  |  |  |
| JS05 | F:CGGCATTACAGTCGGCAGTA | (GAA)10 | 93-120 | 57 | Xu et al. 2020 | TAMRA | M 3 |
|  | R:ACAATTCCCGTGCTGCATCT |  |  |  |  |  |  |
| JS06 | F:CCCTGCATGCAATCAATCACA | (AGT)5 | 96-111 | 55 | Xu et al. 2020 | TAMRA | M 4 |
|  | R:ATGGGACGAGTGATGGACTC |  |  |  |  |  |  |
| JS07 | F:ACCAGCAGTTCCATGTACGG | (GAG)9 | 111-132 | 57 | Xu et al. 2020 | HEX | M 2 |
|  | R:GCTCATGCCATTATCTGCTTCG |  |  |  |  |  |  |
| JS09 | F:TTCGACCGCGTTTCCAGTTA | (TTC)7 | 116-131 | 56 | Xu et al. 2020 | FAM | M 1 |
|  | R:CCAGACTCACGGTCAGTTCC |  |  |  |  |  |  |
| JS12 | F:TCAACATTGGCGAGGTGACA | (TTA)7 | 128-155 | 55 | Xu et al. 2020 | TAMRA | M 1 |
|  | R:AGGCAAGTCTACTTCTTTCCCT |  |  |  |  |  |  |
| JS13 | F:TCTTGTCAGCATACTAAGCTTGTT | (TTCT)5 | 129-158 | 56 | Xu et al. 2020 | HEX | M 5 |
|  | R:ACTAACTGCATATAGGATCAACCA |  |  |  |  |  |  |
| JS14 | F:CACATCGAGTGTTTCAAGTGACA | (TGC)6 | 134-149 | 57 | Xu et al. 2020 | FAM | M 4 |
|  | R:TGCACATGAGGAATTAACTGCTT |  |  |  |  |  |  |
| JS15 | F:ATCTCCGTGACTCCGCTCCT | (TTG)5 | 352-377 | 60 | Xu et al. 2020 | TAMRA | M 3 |
|  | R:ACCCGCCACCATCTTCATCTACCAA |  |  |  |  |  |  |
| JS22 | F:AAAGTTGCTCCTCAGCTTGG | (ATC)7 | 266-293 | 56 | Xu et al. 2020 | FAM | M 4 |
|  | R:TAATTAGCAATGAACAGATGGTGG |  |  |  |  |  |  |
| JS28 | L:AAAGGGTGAAGGAAGAAATTAGGAT | (AAGAG)5 | 316-332 | 57 | Xu et al. 2020 | HEX | M 5 |
|  | R:CCAAATTAAGCCAAACATGGTTGC |  |  |  |  |  |  |
| BFU-Jr277 | F:TATTCACCCGGAGGTTTCAG | (GAT)10 | 235-250 | 61 | Zhang et al. 2013 | FAM | M 3 |
|  | R:CCGAAGCCAGTCGAGTTATC |  |  |  |  |  |  |
| BFU-Jr38 | F:AGCTCCTCAAGCAAGGCTTA | (GAT)13 | 127-145 | 60 | Zhang et al. 2013 | FAM | M 3 |
|  | R:GTGCATGGAACCACACTCAG |  |  |  |  |  |  |
| CUJRD102 | F-GACAGCAGCCTTATTTTGTAAC | (GAG)8 | 169-184 | 53 | Topçu et al. 2015 | HEX | M 4 |
|  | R-TTCGTCCTCTTCTTCTTCAAC |  |  |  |  |  |  |
| CUJRD462 | F-TGCTCATTTTCATCCACTATC | (GAA)8 | 250-268 | 55 | Topçu et al. 2015 | HEX | M 2 |
|  | R-ACTTCCTCTCCTTCCTCTTTC |  |  |  |  |  |  |
| JM5446 | F-ATGCATGCAGCTCCTACCTC | (CTAG)5 | 221-249 | 56 | Hu et al. 2016 | HEX | M 3 |
|  | R-GGACGTGTCCTGGGTTTTCA |  |  |  |  |  |  |
| SSR18 | F:GGAAAGGGATTTGAGGAGAGAT | (TTC)8 | 297-303 | 60 | Huan et al. 2013 | TAMRA | M 5 |
|  | R:GAAGAGGAGGAAGAAGAGGAGG |  |  |  |  |  |  |
| ZMZ7 | F:GAACAAATAGACCAGGCACG | (TCC)7 | 215-236 | 56 | Qi et al. 2009 | TAMRA | M 1 |
|  | R:TAACGACAACCGATGAAACC |  |  |  |  |  |  |
| Note: Multiplexing information, each multiplex system has a consensus annealing temperature, M1 (57℃), M2 (58℃), M3 (56℃), M4 (58℃), M5 (55℃). | | | | | | | |

**Table S3**. Population pairwise *F*_ST_ and Nei’s genetic distance (*D*_A_). Lower-left part: *F*_ST_; upper-right part: *D*_A_.

| Pop. | ARR | ESR | ETR | GDR | KNR | QAR | RGR | RUR | SAR | TER | WER | ZAR | AKR | EHR | GSR | HMR | JBR | KDR | KHR | KSR | MZR | NGR | TAR | TKR | TPR | SMR | GMR |
| --- | --- | --- | --- | --- | --- | --- | --- | --- | --- | --- | --- | --- | --- | --- | --- | --- | --- | --- | --- | --- | --- | --- | --- | --- | --- | --- | --- |
| ARR | 0.0000 | 0.0503 | 0.0617 | 0.1201 | 0.0871 | 0.1454 | 0.1276 | 0.1338 | 0.1408 | 0.1448 | 0.0941 | 0.1082 | 0.1046 | 0.1031 | 0.1513 | 0.1119 | 0.1030 | 0.1342 | 0.0998 | 0.1467 | 0.1406 | 0.1160 | 0.1545 | 0.2004 | 0.1467 | 0.1345 | 0.1454 |
| ESR | 0.0464 | 0.0000 | 0.0468 | 0.1355 | 0.0888 | 0.1430 | 0.1239 | 0.1335 | 0.1272 | 0.1371 | 0.0683 | 0.0835 | 0.0941 | 0.0830 | 0.1337 | 0.0980 | 0.1011 | 0.1082 | 0.0830 | 0.1167 | 0.1212 | 0.0998 | 0.1467 | 0.1545 | 0.1426 | 0.1177 | 0.1611 |
| ETR | 0.0638 | 0.0585 | 0.0000 | 0.1490 | 0.1067 | 0.1632 | 0.1278 | 0.1466 | 0.1385 | 0.1538 | 0.0822 | 0.0774 | 0.0864 | 0.0941 | 0.1405 | 0.0928 | 0.0904 | 0.1085 | 0.1042 | 0.1195 | 0.1492 | 0.0984 | 0.1597 | 0.1806 | 0.1504 | 0.1157 | 0.1534 |
| GDR | 0.1190 | 0.1463 | 0.1477 | 0.0000 | 0.1304 | 0.1208 | 0.1327 | 0.1303 | 0.0812 | 0.1120 | 0.1295 | 0.1307 | 0.1276 | 0.1303 | 0.1529 | 0.1247 | 0.1047 | 0.1441 | 0.1273 | 0.1379 | 0.1384 | 0.1349 | 0.1321 | 0.1778 | 0.1371 | 0.1266 | 0.1411 |
| KNR | 0.0509 | 0.0770 | 0.1167 | 0.1064 | 0.0000 | 0.1164 | 0.1052 | 0.1101 | 0.1286 | 0.1400 | 0.0909 | 0.1241 | 0.1144 | 0.0960 | 0.1179 | 0.1130 | 0.0930 | 0.1135 | 0.1021 | 0.1146 | 0.1239 | 0.1080 | 0.1251 | 0.1615 | 0.1323 | 0.1157 | 0.1284 |
| QAR | 0.1635 | 0.1640 | 0.1996 | 0.1184 | 0.1383 | 0.0000 | 0.1029 | 0.0851 | 0.0982 | 0.0694 | 0.1260 | 0.1483 | 0.0986 | 0.1156 | 0.1107 | 0.1252 | 0.0971 | 0.1320 | 0.1261 | 0.1463 | 0.0999 | 0.1394 | 0.1275 | 0.1308 | 0.0976 | 0.1192 | 0.1609 |
| RGR | 0.1239 | 0.1334 | 0.1326 | 0.1068 | 0.1111 | 0.0933 | 0.0000 | 0.0742 | 0.1321 | 0.1369 | 0.1125 | 0.1344 | 0.0977 | 0.1226 | 0.1248 | 0.1069 | 0.0876 | 0.1436 | 0.1145 | 0.1600 | 0.1072 | 0.1099 | 0.1229 | 0.1510 | 0.1203 | 0.1328 | 0.1957 |
| RUR | 0.1386 | 0.1260 | 0.1628 | 0.1287 | 0.1141 | 0.0903 | 0.0716 | 0.0000 | 0.1162 | 0.1241 | 0.1307 | 0.1293 | 0.1159 | 0.1285 | 0.1175 | 0.0891 | 0.0919 | 0.0989 | 0.1252 | 0.1374 | 0.1059 | 0.1257 | 0.1262 | 0.1148 | 0.1094 | 0.1066 | 0.1528 |
| SAR | 0.1486 | 0.1310 | 0.1497 | 0.0506 | 0.1410 | 0.1172 | 0.1284 | 0.1166 | 0.0000 | 0.1009 | 0.1096 | 0.1163 | 0.0913 | 0.1134 | 0.1183 | 0.0941 | 0.0779 | 0.0983 | 0.1237 | 0.1038 | 0.1380 | 0.1129 | 0.1290 | 0.1613 | 0.1193 | 0.1105 | 0.1562 |
| TER | 0.1847 | 0.1726 | 0.1916 | 0.1235 | 0.1614 | 0.0857 | 0.1594 | 0.1404 | 0.1050 | 0.0000 | 0.1252 | 0.1385 | 0.1211 | 0.1126 | 0.1153 | 0.1232 | 0.0961 | 0.0982 | 0.1306 | 0.1215 | 0.1077 | 0.1413 | 0.1303 | 0.1194 | 0.1123 | 0.1008 | 0.1526 |
| WER | 0.0670 | 0.0499 | 0.0658 | 0.0946 | 0.0703 | 0.1374 | 0.0978 | 0.1252 | 0.1057 | 0.1359 | 0.0000 | 0.0862 | 0.0826 | 0.0806 | 0.1272 | 0.0685 | 0.0786 | 0.1101 | 0.0909 | 0.0978 | 0.1290 | 0.0996 | 0.1053 | 0.1292 | 0.1058 | 0.0944 | 0.1377 |
| ZAR | 0.1284 | 0.0930 | 0.0853 | 0.1405 | 0.1577 | 0.1998 | 0.1710 | 0.1654 | 0.1264 | 0.1803 | 0.1049 | 0.0000 | 0.0813 | 0.1018 | 0.1334 | 0.0783 | 0.0906 | 0.1043 | 0.1039 | 0.1025 | 0.1549 | 0.0927 | 0.1490 | 0.1443 | 0.1373 | 0.0935 | 0.1370 |
| AKR | 0.0966 | 0.0853 | 0.0791 | 0.0886 | 0.1024 | 0.0849 | 0.0807 | 0.0944 | 0.0828 | 0.1052 | 0.0593 | 0.0989 | 0.0000 | 0.0747 | 0.0934 | 0.0661 | 0.0598 | 0.0894 | 0.0753 | 0.1235 | 0.1275 | 0.0646 | 0.1160 | 0.1364 | 0.1011 | 0.1071 | 0.1395 |
| EHR | 0.0892 | 0.0799 | 0.0751 | 0.1030 | 0.0859 | 0.1148 | 0.1059 | 0.1283 | 0.1108 | 0.1000 | 0.0609 | 0.1120 | 0.0459 | 0.0000 | 0.1166 | 0.0889 | 0.0619 | 0.0864 | 0.0675 | 0.0982 | 0.1120 | 0.0804 | 0.1156 | 0.1247 | 0.1154 | 0.1073 | 0.1487 |
| GSR | 0.1617 | 0.1507 | 0.1564 | 0.1483 | 0.1320 | 0.1122 | 0.1315 | 0.1109 | 0.1352 | 0.1109 | 0.1284 | 0.1696 | 0.0740 | 0.1045 | 0.0000 | 0.1337 | 0.0992 | 0.0898 | 0.1515 | 0.1200 | 0.1209 | 0.1163 | 0.1431 | 0.1483 | 0.1234 | 0.0916 | 0.1451 |
| HMR | 0.1113 | 0.0897 | 0.0993 | 0.1160 | 0.1108 | 0.1440 | 0.0973 | 0.0781 | 0.1022 | 0.1455 | 0.0498 | 0.0970 | 0.0593 | 0.0737 | 0.1449 | 0.0000 | 0.0606 | 0.0799 | 0.0932 | 0.0952 | 0.1314 | 0.0852 | 0.0901 | 0.1086 | 0.0870 | 0.0914 | 0.1469 |
| JBR | 0.1124 | 0.1259 | 0.0987 | 0.0881 | 0.1168 | 0.1095 | 0.0871 | 0.0949 | 0.0780 | 0.0952 | 0.0825 | 0.1068 | 0.0440 | 0.0499 | 0.1240 | 0.0510 | 0.0000 | 0.0752 | 0.0589 | 0.0860 | 0.1051 | 0.0609 | 0.0981 | 0.1244 | 0.0941 | 0.0861 | 0.1439 |
| KDR | 0.1784 | 0.1327 | 0.1373 | 0.1574 | 0.1572 | 0.1759 | 0.1810 | 0.1349 | 0.1175 | 0.1000 | 0.1230 | 0.1271 | 0.0942 | 0.0840 | 0.0969 | 0.1069 | 0.0964 | 0.0000 | 0.1174 | 0.0749 | 0.1395 | 0.1072 | 0.1381 | 0.1252 | 0.1149 | 0.0657 | 0.1430 |
| KHR | 0.0974 | 0.0785 | 0.1261 | 0.1109 | 0.1016 | 0.1249 | 0.1134 | 0.1347 | 0.1226 | 0.1347 | 0.0899 | 0.1328 | 0.0767 | 0.0660 | 0.1790 | 0.0909 | 0.0711 | 0.1623 | 0.0000 | 0.1103 | 0.1197 | 0.0765 | 0.1193 | 0.1448 | 0.1233 | 0.1212 | 0.1590 |
| KSR | 0.1647 | 0.1143 | 0.1295 | 0.1304 | 0.1390 | 0.1737 | 0.1683 | 0.1573 | 0.1001 | 0.1246 | 0.0914 | 0.1010 | 0.1147 | 0.0847 | 0.1186 | 0.1025 | 0.1029 | 0.0703 | 0.1318 | 0.0000 | 0.1319 | 0.1084 | 0.1323 | 0.1440 | 0.1193 | 0.0718 | 0.1527 |
| MZR | 0.1416 | 0.1251 | 0.1653 | 0.1318 | 0.1300 | 0.0943 | 0.0922 | 0.1051 | 0.1428 | 0.1387 | 0.1302 | 0.1935 | 0.1072 | 0.1139 | 0.1192 | 0.1230 | 0.1379 | 0.1842 | 0.1325 | 0.1384 | 0.0000 | 0.1256 | 0.1212 | 0.1122 | 0.0940 | 0.1313 | 0.1692 |
| NGR | 0.0984 | 0.0943 | 0.0978 | 0.1106 | 0.0942 | 0.1348 | 0.1005 | 0.1209 | 0.1028 | 0.1506 | 0.0921 | 0.1062 | 0.0489 | 0.0652 | 0.1263 | 0.0708 | 0.0527 | 0.1125 | 0.0703 | 0.0965 | 0.1156 | 0.0000 | 0.1246 | 0.1530 | 0.1158 | 0.1078 | 0.1644 |
| TAR | 0.1990 | 0.1982 | 0.2041 | 0.1407 | 0.1507 | 0.1827 | 0.1509 | 0.1686 | 0.1505 | 0.1772 | 0.1213 | 0.2136 | 0.1437 | 0.1430 | 0.1818 | 0.0965 | 0.1346 | 0.1855 | 0.1655 | 0.1463 | 0.1710 | 0.1620 | 0.0000 | 0.1274 | 0.0491 | 0.1294 | 0.1561 |
| TKR | 0.2259 | 0.1713 | 0.2135 | 0.1878 | 0.1903 | 0.1383 | 0.1530 | 0.1200 | 0.1774 | 0.1478 | 0.1413 | 0.2181 | 0.1161 | 0.1482 | 0.1567 | 0.1092 | 0.1638 | 0.1629 | 0.1722 | 0.1726 | 0.1166 | 0.1694 | 0.1777 | 0.0000 | 0.1102 | 0.1259 | 0.1786 |
| TPR | 0.1916 | 0.1823 | 0.1853 | 0.1492 | 0.1590 | 0.1324 | 0.1436 | 0.1428 | 0.1377 | 0.1438 | 0.1245 | 0.1947 | 0.1053 | 0.1290 | 0.1280 | 0.1006 | 0.1260 | 0.1351 | 0.1832 | 0.1245 | 0.1250 | 0.1298 | 0.0426 | 0.1348 | 0.0000 | 0.1030 | 0.1521 |
| SMR | 0.1778 | 0.1456 | 0.1525 | 0.1494 | 0.1641 | 0.1712 | 0.1749 | 0.1490 | 0.1184 | 0.1089 | 0.1054 | 0.1205 | 0.1154 | 0.1129 | 0.1024 | 0.1224 | 0.1040 | 0.0531 | 0.1688 | 0.0583 | 0.1941 | 0.1316 | 0.1747 | 0.1859 | 0.1308 | 0.0000 | 0.1455 |
| GMR | 0.1268 | 0.1402 | 0.1270 | 0.0889 | 0.0910 | 0.1507 | 0.1326 | 0.1343 | 0.1193 | 0.1441 | 0.0744 | 0.1465 | 0.0671 | 0.0912 | 0.1175 | 0.1005 | 0.1147 | 0.1294 | 0.1396 | 0.1530 | 0.1539 | 0.1251 | 0.1330 | 0.1747 | 0.1273 | 0.1611 | 0.0000 |

Note: The values of *F*_ST_ ranged from 0 to 1. The level of genetic differentiation refers to Wright (1978).

Reference: Wright, S. (1978). Evolution and the Genetics of Populations. Chicago, IL, USA: The University of Chicago Press.

| **Table S4.** Gene flow (*N*_m_) among populations. | | | | | | | | | | | | | | | | | | | | | | | | | | | |
| --- | --- | --- | --- | --- | --- | --- | --- | --- | --- | --- | --- | --- | --- | --- | --- | --- | --- | --- | --- | --- | --- | --- | --- | --- | --- | --- | --- |
| Pop. | ARR | ESR | ETR | GDR | KNR | QAR | RGR | RUR | SAR | TER | WER | ZAR | AKR | EHR | GSR | HMR | JBR | KDR | KHR | KSR | MZR | NGR | TAR | TKR | TPR | SMR | GMR |
| ARR | 0.000 |  |  |  |  |  |  |  |  |  |  |  |  |  |  |  |  |  |  |  |  |  |  |  |  |  |  |
| ESR | 5.134 | 0.000 |  |  |  |  |  |  |  |  |  |  |  |  |  |  |  |  |  |  |  |  |  |  |  |  |  |
| ETR | 3.670 | 4.026 | 0.000 |  |  |  |  |  |  |  |  |  |  |  |  |  |  |  |  |  |  |  |  |  |  |  |  |
| GDR | 1.852 | 1.459 | 1.443 | 0.000 |  |  |  |  |  |  |  |  |  |  |  |  |  |  |  |  |  |  |  |  |  |  |  |
| KNR | 4.664 | 2.997 | 1.892 | 2.100 | 0.000 |  |  |  |  |  |  |  |  |  |  |  |  |  |  |  |  |  |  |  |  |  |  |
| QAR | 1.279 | 1.274 | 1.002 | 1.861 | 1.558 | 0.000 |  |  |  |  |  |  |  |  |  |  |  |  |  |  |  |  |  |  |  |  |  |
| RGR | 1.768 | 1.624 | 1.635 | 2.090 | 2.001 | 2.431 | 0.000 |  |  |  |  |  |  |  |  |  |  |  |  |  |  |  |  |  |  |  |  |
| RUR | 1.553 | 1.734 | 1.285 | 1.693 | 1.942 | 2.518 | 3.243 | 0.000 |  |  |  |  |  |  |  |  |  |  |  |  |  |  |  |  |  |  |  |
| SAR | 1.432 | 1.658 | 1.420 | 4.688 | 1.524 | 1.883 | 1.697 | 1.894 | 0.000 |  |  |  |  |  |  |  |  |  |  |  |  |  |  |  |  |  |  |
| TER | 1.104 | 1.198 | 1.055 | 1.775 | 1.299 | 2.667 | 1.318 | 1.531 | 2.131 | 0.000 |  |  |  |  |  |  |  |  |  |  |  |  |  |  |  |  |  |
| WER | 3.481 | 4.765 | 3.551 | 2.392 | 3.309 | 1.570 | 2.306 | 1.747 | 2.115 | 1.589 | 0.000 |  |  |  |  |  |  |  |  |  |  |  |  |  |  |  |  |
| ZAR | 1.697 | 2.438 | 2.680 | 1.530 | 1.335 | 1.001 | 1.212 | 1.261 | 1.728 | 1.137 | 2.132 | 0.000 |  |  |  |  |  |  |  |  |  |  |  |  |  |  |  |
| AKR | 2.339 | 2.682 | 2.910 | 2.571 | 2.191 | 2.694 | 2.849 | 2.400 | 2.770 | 2.126 | 3.967 | 2.278 | 0.000 |  |  |  |  |  |  |  |  |  |  |  |  |  |  |
| EHR | 2.551 | 2.879 | 3.080 | 2.178 | 2.662 | 1.929 | 2.112 | 1.698 | 2.007 | 2.250 | 3.858 | 1.983 | 5.199 | 0.000 |  |  |  |  |  |  |  |  |  |  |  |  |  |
| GSR | 1.296 | 1.409 | 1.348 | 1.436 | 1.643 | 1.978 | 1.651 | 2.005 | 1.599 | 2.005 | 1.697 | 1.224 | 3.129 | 2.142 | 0.000 |  |  |  |  |  |  |  |  |  |  |  |  |
| HMR | 1.996 | 2.537 | 2.268 | 1.905 | 2.006 | 1.487 | 2.320 | 2.949 | 2.196 | 1.469 | 4.770 | 2.328 | 3.964 | 3.144 | 1.476 | 0.000 |  |  |  |  |  |  |  |  |  |  |  |
| JBR | 1.973 | 1.735 | 2.284 | 2.588 | 1.890 | 2.033 | 2.621 | 2.384 | 2.953 | 2.377 | 2.780 | 2.092 | 5.432 | 4.758 | 1.765 | 4.651 | 0.000 |  |  |  |  |  |  |  |  |  |  |
| KDR | 1.152 | 1.634 | 1.571 | 1.338 | 1.340 | 1.171 | 1.131 | 1.603 | 1.878 | 2.250 | 1.782 | 1.717 | 2.405 | 2.725 | 2.331 | 2.088 | 2.342 | 0.000 |  |  |  |  |  |  |  |  |  |
| KHR | 2.318 | 2.935 | 1.733 | 2.005 | 2.210 | 1.751 | 1.954 | 1.605 | 1.790 | 1.607 | 2.531 | 1.632 | 3.009 | 3.541 | 1.146 | 2.501 | 3.265 | 1.290 | 0.000 |  |  |  |  |  |  |  |  |
| KSR | 1.268 | 1.937 | 1.681 | 1.668 | 1.548 | 1.189 | 1.236 | 1.339 | 2.247 | 1.757 | 2.486 | 2.225 | 1.930 | 2.702 | 1.858 | 2.190 | 2.179 | 3.307 | 1.647 | 0.000 |  |  |  |  |  |  |  |
| MZR | 1.515 | 1.749 | 1.262 | 1.648 | 1.673 | 2.400 | 2.462 | 2.128 | 1.500 | 1.552 | 1.671 | 1.042 | 2.083 | 1.945 | 1.848 | 1.782 | 1.562 | 1.107 | 1.637 | 1.556 | 0.000 |  |  |  |  |  |  |
| NGR | 2.290 | 2.402 | 2.306 | 2.010 | 2.403 | 1.604 | 2.238 | 1.818 | 2.182 | 1.410 | 2.465 | 2.104 | 4.858 | 3.583 | 1.729 | 3.283 | 4.493 | 1.973 | 3.305 | 2.341 | 1.913 | 0.000 |  |  |  |  |  |
| TAR | 1.006 | 1.011 | 0.975 | 1.527 | 1.409 | 1.119 | 1.407 | 1.233 | 1.411 | 1.161 | 1.812 | 0.920 | 1.490 | 1.498 | 1.125 | 2.340 | 1.607 | 1.098 | 1.261 | 1.459 | 1.212 | 1.293 | 0.000 |  |  |  |  |
| TKR | 0.857 | 1.209 | 0.921 | 1.081 | 1.063 | 1.557 | 1.384 | 1.833 | 1.159 | 1.442 | 1.519 | 0.896 | 1.903 | 1.437 | 1.345 | 2.039 | 1.277 | 1.285 | 1.202 | 1.198 | 1.894 | 1.226 | 1.157 | 0.000 |  |  |  |
| TPR | 1.055 | 1.121 | 1.099 | 1.425 | 1.323 | 1.639 | 1.490 | 1.501 | 1.565 | 1.489 | 1.758 | 1.034 | 2.124 | 1.688 | 1.703 | 2.235 | 1.734 | 1.601 | 1.115 | 1.758 | 1.750 | 1.676 | 5.614 | 1.605 | 0.000 |  |  |
| SMR | 1.156 | 1.467 | 1.390 | 1.423 | 1.273 | 1.210 | 1.180 | 1.428 | 1.861 | 2.045 | 2.123 | 1.826 | 1.917 | 1.964 | 2.191 | 1.793 | 2.155 | 4.461 | 1.231 | 4.036 | 1.038 | 1.650 | 1.181 | 1.095 | 1.661 | 0.000 |  |
| GMR | 1.721 | 1.533 | 1.719 | 2.562 | 2.498 | 1.409 | 1.636 | 1.612 | 1.845 | 1.485 | 3.110 | 1.457 | 3.478 | 2.492 | 1.878 | 2.238 | 1.930 | 1.682 | 1.540 | 1.384 | 1.374 | 1.749 | 1.630 | 1.181 | 1.714 | 1.302 | 0.000 |

**
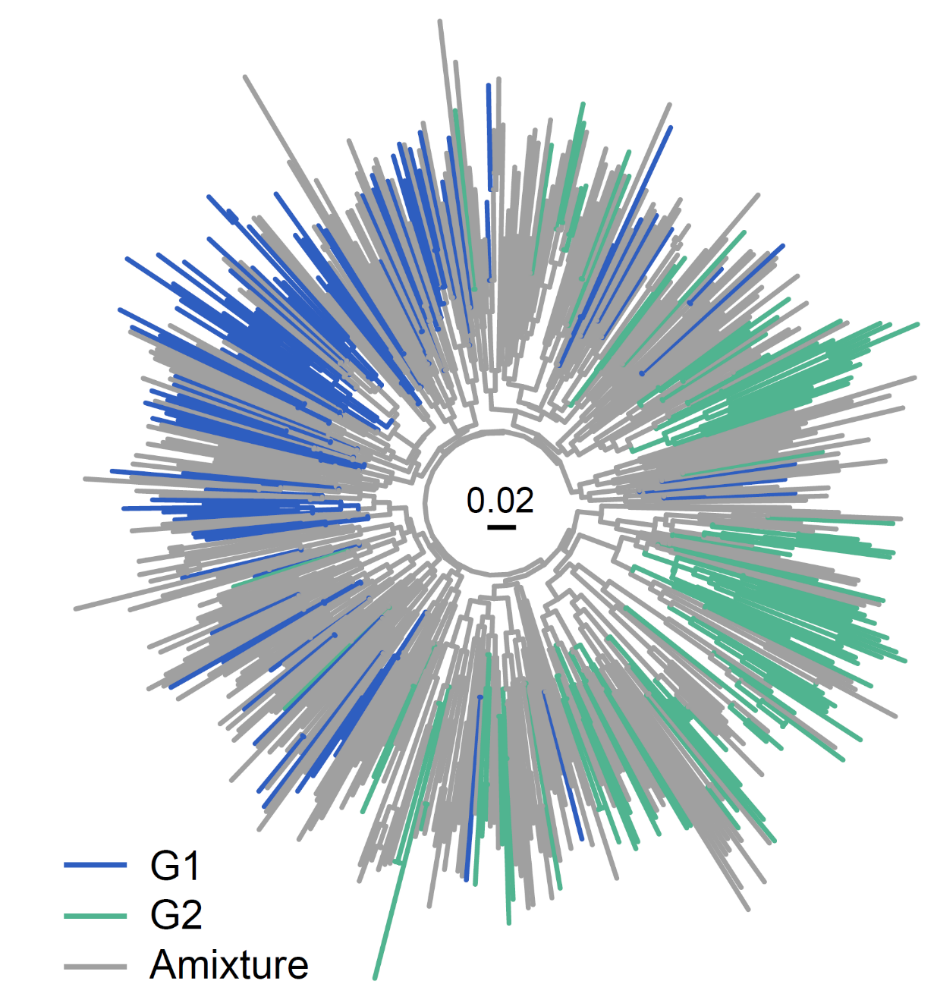
**

**Fig. S1.** Neighbor-joining tree of 508 individuals, colors correspond to figure 4. The designation of G1 and G2 corresponds to Fig. 2a, while the grey branches represent mixed individuals.


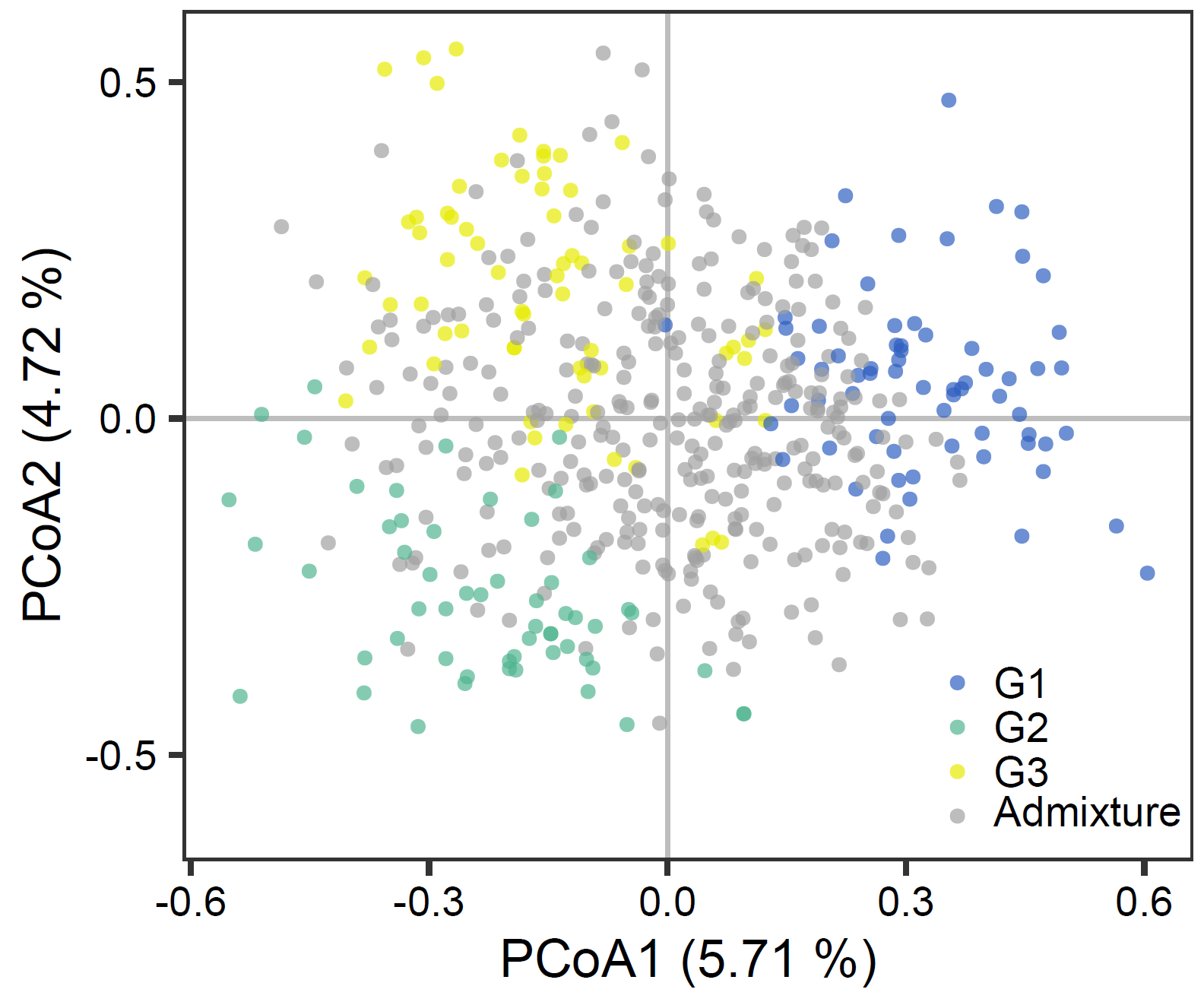


**Fig. S2.** Principal Co-ordinates Analysis (PCoA) analysis of 508 individuals based on Nei’s genetic distance (*D*_A_). The groups were defined according to STRUCTURE analysis (*K =* 3, *Q* > 0.8).


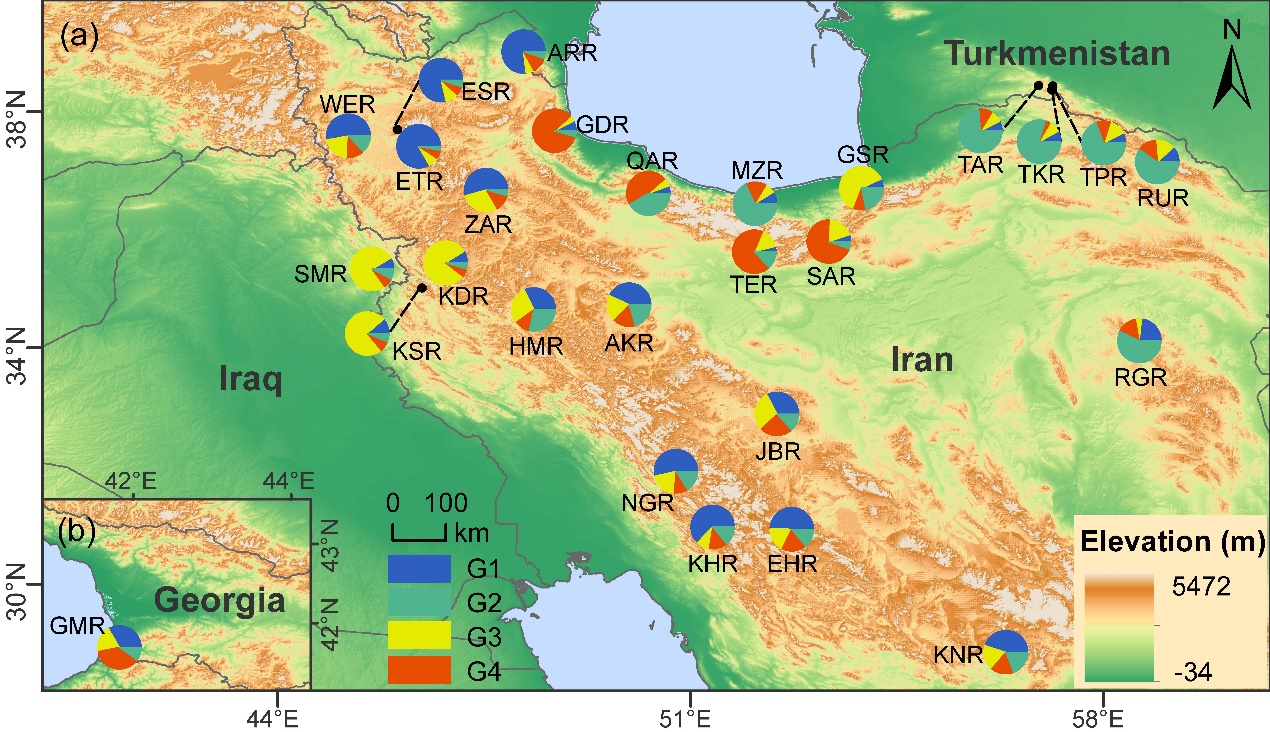


**Fig. S3.** Geographical distribution of the genetic structure of 27 populations. Pie charts show the genetic proposition of each cluster in STRUCTURE analysis (*K* = 4).


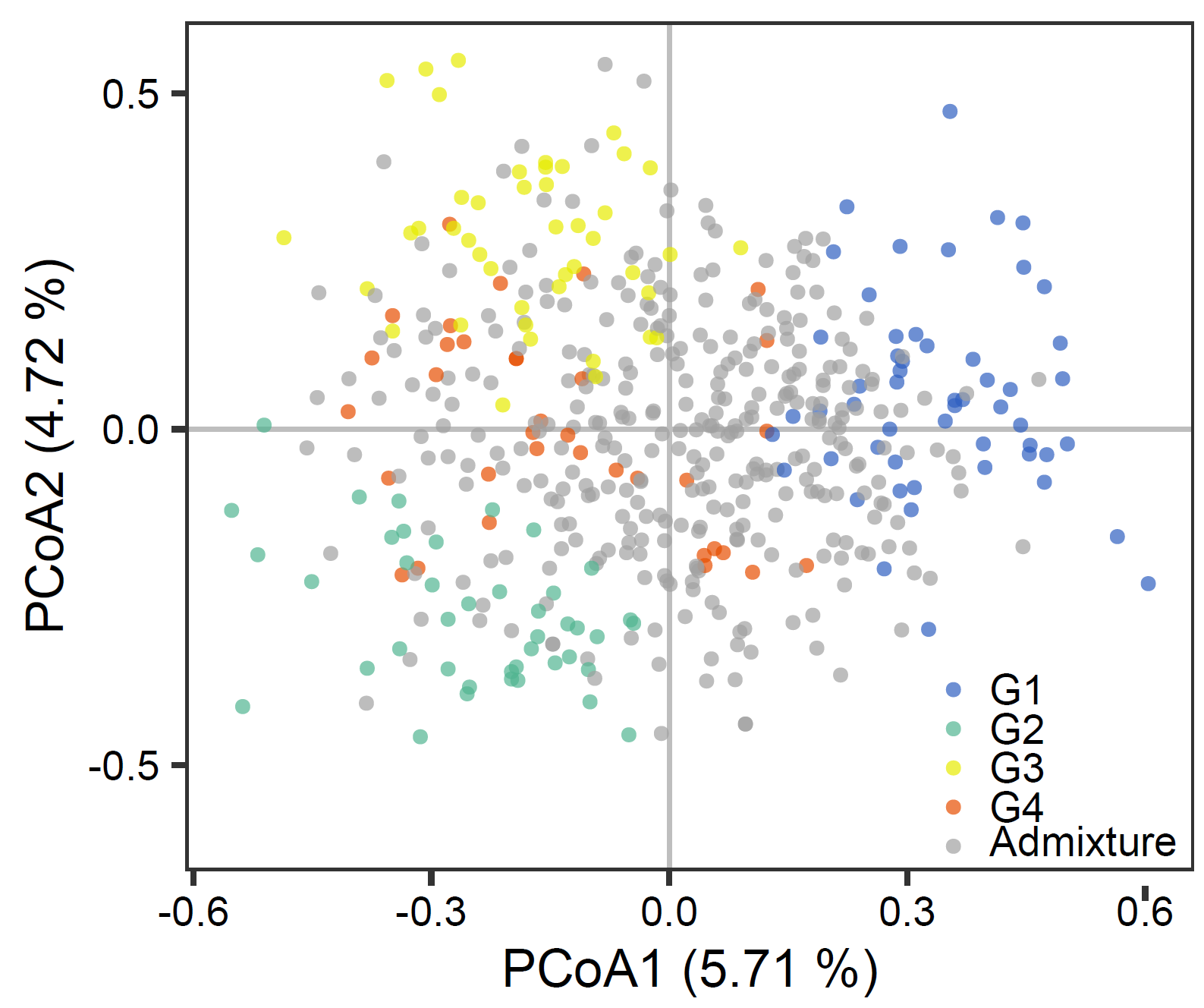


**Fig. S4.** Principal Co-ordinates Analysis (PCoA) of 508 individuals based on Nei’s genetic distance (*D*_A_). The groups were defined according to STRUCTURE analysis (*K =* 4, *Q* > 0.8).


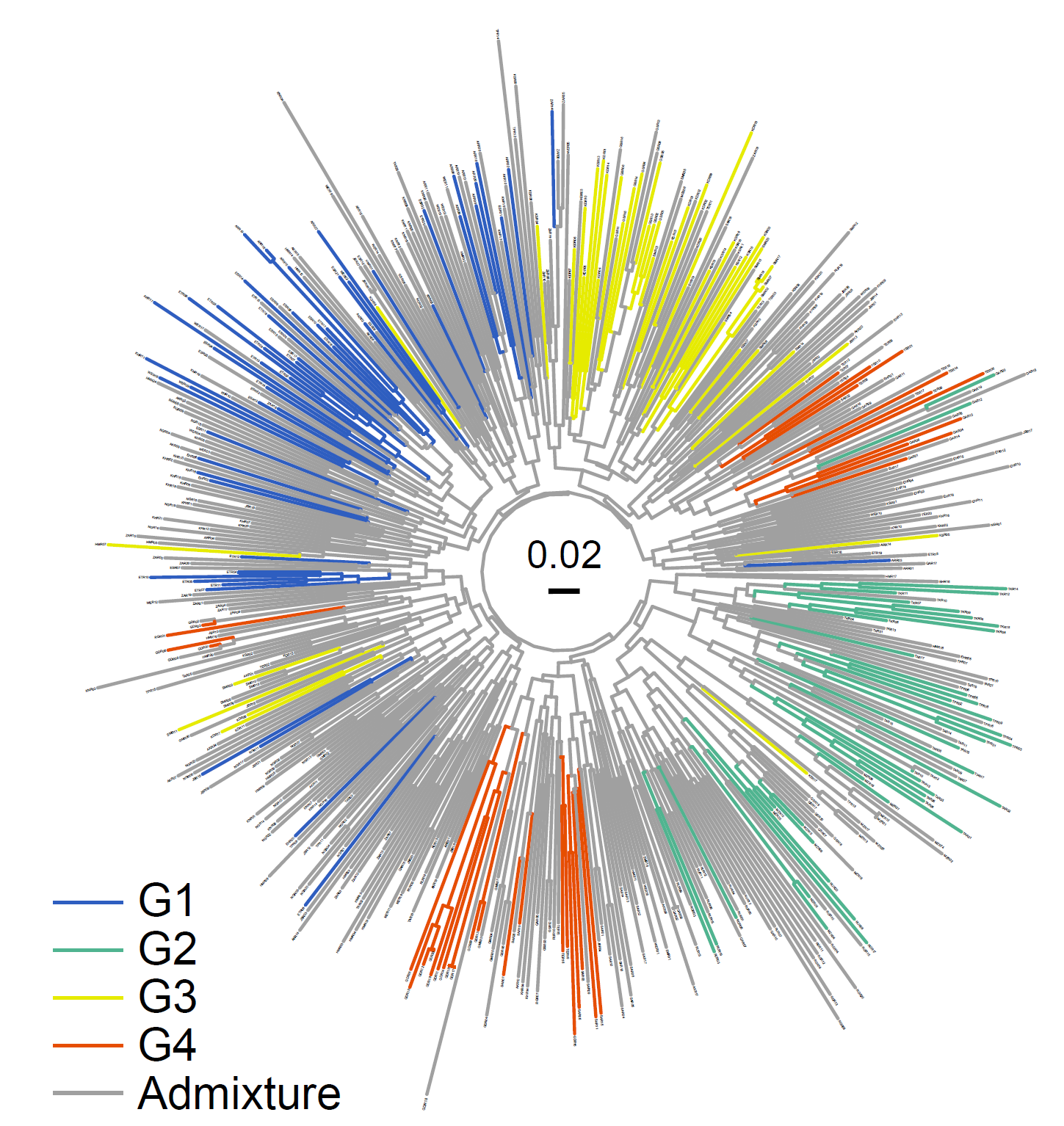


**F****ig. S5.** Neighbor-joining tree of 508 individuals. Colors correspond to Fig. S4.


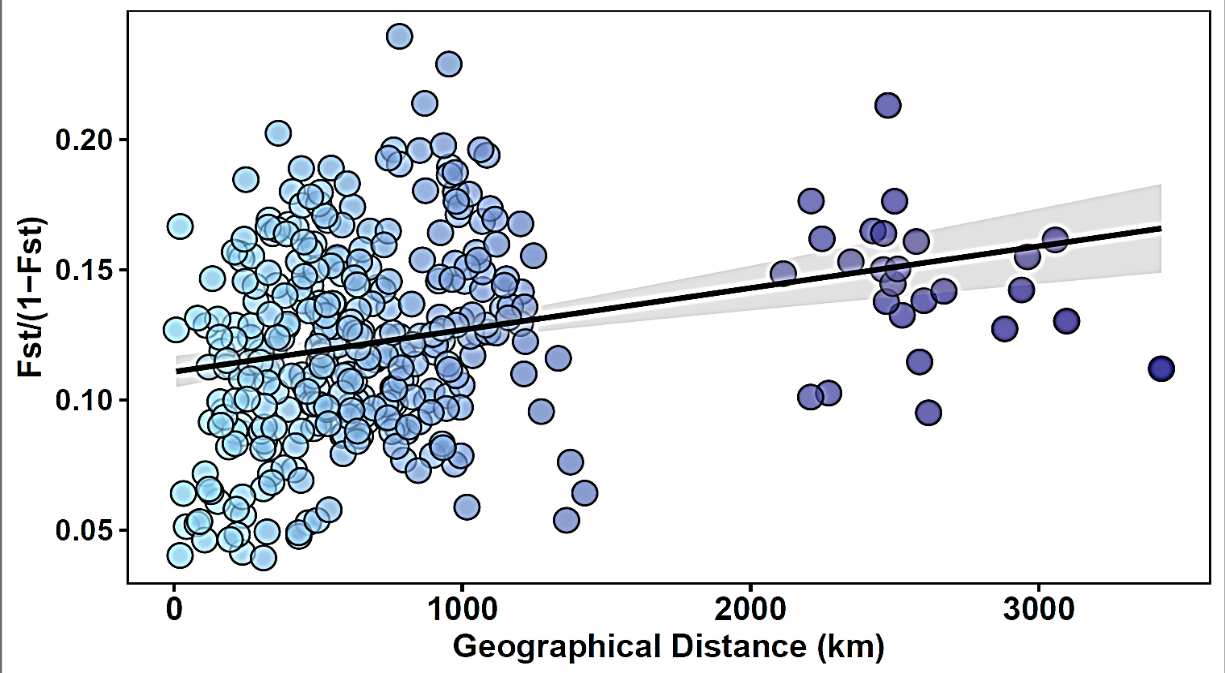


**(a)**


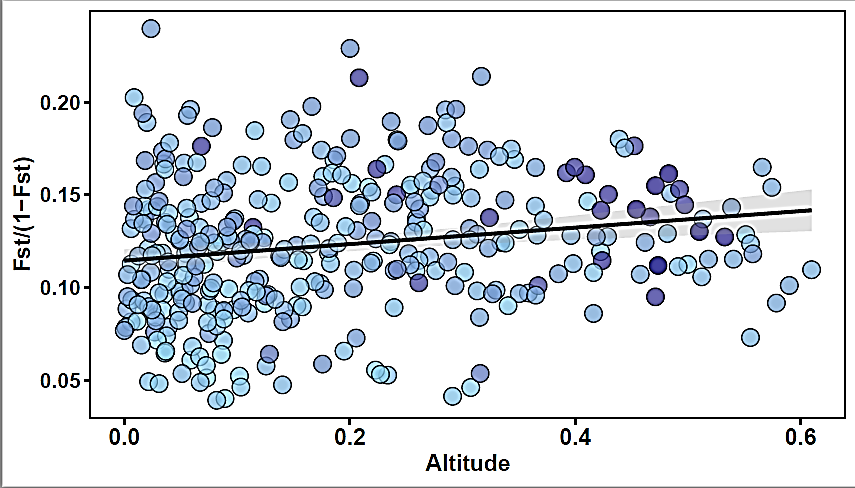


**(b)**

**(m)**

***F*_ST_/(1-*F*_ST_)**

***F*_ST_/(1-*F*_ST_)**

**Fig. S6.** Genetic isolation by distance of 27 populations in the Iranian Plateau. **(a)** genetic distance and geographical distance (*r* = 0.26, *P* = 0.05), **(b)** genetic distance and altitude (*r*= -0.19, *P*= 0.05).
